# Genomics, social media and mobile phone data enable mapping of SARS-CoV-2 lineages to inform health policy in Bangladesh

**DOI:** 10.1038/s41564-021-00955-3

**Published:** 2021-09-08

**Authors:** Lauren A. Cowley, Mokibul Hassan Afrad, Sadia Isfat Ara Rahman, Md Mahfuz Al Mamun, Taylor Chin, Ayesha Mahmud, Mohammed Ziaur Rahman, Mallick Masum Billah, Manjur Hossain Khan, Sharmin Sultana, Tilovatul Khondaker, Stephen Baker, Nandita Banik, Ahmed Nawsher Alam, Kaiissar Mannoor, Sayera Banu, Anir Chowdhury, Meerjady Sabrina Flora, Nicholas R. Thomson, Caroline O. Buckee, Firdausi Qadri, Tahmina Shirin

**Affiliations:** 1 Department of Biology and Biochemistry, University of Bath, Bath, UK; 2 Infectious Diseases Division, International Centre for Diarrheal Disease Research Bangladesh (icddr,b), Dhaka, Bangladesh; 3 Institute for Developing Science and Health Initiatives, Dhaka, Bangladesh; 4 Center for Communicable Disease Dynamics, Department of Epidemiology, Harvard T. H. Chan School of Public Health, Boston, MA USA; 5 Department of Demography, University of California, Berkeley, CA USA; 6 Institute of Epidemiology, Disease Control and Research (IEDCR), Dhaka, Bangladesh; 7 Department of Medicine, University of Cambridge, Cambridge, UK; 8 Aspire to Innovate (a2i) Program, ICT Division/Cabinet Division, Government of Bangladesh/UNDP, Dhaka, Bangladesh; 9 Directorate General of Health Services, Mohakhali, Dhaka, Bangladesh; 10 Wellcome Sanger Institute, Wellcome Genome Campus, Hinxton, UK; 11 London School of Hygiene and Tropical Medicine, London, UK

**Keywords:** Policy and public health in microbiology, Viral genetics

## Abstract

Genomics, combined with population mobility data, used to map importation and spatial spread of SARS-CoV-2 in high-income countries has enabled the implementation of local control measures. Here, to track the spread of SARS-CoV-2 lineages in Bangladesh at the national level, we analysed outbreak trajectory and variant emergence using genomics, Facebook ‘Data for Good’ and data from three mobile phone operators. We sequenced the complete genomes of 67 SARS-CoV-2 samples (collected by the IEDCR in Bangladesh between March and July 2020) and combined these data with 324 publicly available Global Initiative on Sharing All Influenza Data (GISAID) SARS-CoV-2 genomes from Bangladesh at that time. We found that most (85%) of the sequenced isolates were Pango lineage B.1.1.25 (58%), B.1.1 (19%) or B.1.36 (8%) in early-mid 2020. Bayesian time-scaled phylogenetic analysis predicted that SARS-CoV-2 first emerged during mid-February in Bangladesh, from abroad, with the first case of coronavirus disease 2019 (COVID-19) reported on 8 March 2020. At the end of March 2020, three discrete lineages expanded and spread clonally across Bangladesh. The shifting pattern of viral diversity in Bangladesh, combined with the mobility data, revealed that the mass migration of people from cities to rural areas at the end of March, followed by frequent travel between Dhaka (the capital of Bangladesh) and the rest of the country, disseminated three dominant viral lineages. Further analysis of an additional 85 genomes (November 2020 to April 2021) found that importation of variant of concern Beta (B.1.351) had occurred and that Beta had become dominant in Dhaka. Our interpretation that population mobility out of Dhaka, and travel from urban hotspots to rural areas, disseminated lineages in Bangladesh in the first wave continues to inform government policies to control national case numbers by limiting within-country travel.

## Abstract (web summary)

In Bangladesh, genomics, social media and mobile phone data streams are integrated to map the spread of SARS-CoV-2 lineages and inform country-level policies to curb infection rates.

## Main

The COVID-19 pandemic has motivated countries around the world to obtain high-resolution data on the local spread of SARS-CoV-2 and arising variants of interest and variants of concern (VOCs). Worldwide, more than 2 million strains have been sequenced and genome information has been made available at GISAID. Within the first 100 days of the emergence of SARS-CoV-2, genomic analyses from various countries led to the development of vaccines that have now reached the market. Genomic surveillance of SARS-CoV-2 is commonplace in high-income countries but is also highly necessary in low- and middle-income countries (LMICs), including Bangladesh, to guide within-country health policies pertinent to the pandemic.

In an unprecedented global response to the COVID-19 pandemic, many countries, including Bangladesh, acted rapidly to restrict population movement and introduce additional social and behavioural interventions to slow the spread of the virus. Until now, the impact of these policies has been hard to assess, in part because of the near-universal difficulties that countries have had rapidly scaling up PCR with reverse transcription (RT–PCR) testing capacity. Bangladesh is a LMIC with a population of more than 166 million people, 63% of whom live in rural regions^1,2^. The first confirmed case of SARS-CoV-2 in Bangladesh was reported on 8 March 2020 by the Institute of Epidemiology Disease Control and Research (IEDCR). To reduce community transmission during the first wave, the Government of Bangladesh announced an official National General Holiday on 23 March effective from 26 March to 4 April, which was thereafter incrementally extended until 30 May. As of 12 July 2021, there were >1 million confirmed COVID-19 cases (case fatality rate, 1.61%)^3^. Although testing capacity was rapidly expanded, training and infrastructure for accurate epidemiological surveillance, particularly outside the capital Dhaka, remained challenging. As a result, it has been unclear how the epidemic has spread in Bangladesh and what this means for the potential spread of SARS-CoV-2 variants, and the best use of interventions, including therapeutics and vaccines.

Viral genomics analyses have been used to track the SARS-CoV-2 epidemic, enabling fine-scaled transmission mapping and analysis of how changes in population behaviour impact patterns of transmission^4^. For example, genomics analysis has been used to disentangle the timeline of spread in Europe^5^ and to understand community transmission versus international importations in New Zealand^6^. Despite sequencing of >2,000 SARS-CoV-2 genomes from Bangladesh, only a handful of studies report the phylogenetic placement of those strains^7–10^.These phylogenetic approaches provide a window into the biology of transmission that is independent of the capacity of the health system to detect cases. Similarly, mobile phone data have been used extensively as a way to monitor the population behavioural response to the epidemic in real time, and to understand the human drivers of transmission^11,12^. These new data streams may be particularly powerful tools for monitoring the pandemic in LMICs, in which RT–PCR testing capacity is often highly constrained.

Worldwide genomic surveillance has been rapidly scaled up in response to the emergence of VOCs in late 2020. Pango lineages^4^ B.1.1.7, B.1.351, P.1 and B.1.617.2 have been designated as VOCs on the basis of their observed heightened risk in terms of transmissibility, severity, vaccine or immune evasion, and have been renamed Alpha, Beta, Gamma and Delta, respectively. It is essential that these variants and any newly emerging variants are observed and monitored by genomic surveillance in LMICs as well as in high-income countries (HICs). This is partly enabled through international data sharing on global databases such as GISAID.

To inform health policies for SARS-CoV-2 control at the national level, we combined viral genomics and population mobility data to analyse the emergence and outbreak trajectory of SARS-CoV-2 in Bangladesh.

## Results

### SARS-CoV-2 lineages in Bangladesh between March and July 2020

**Fig. 1:**
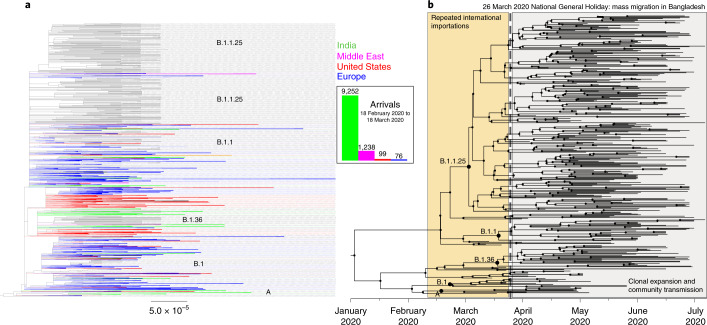
Phylogenetic analysis of 391 Bangladesh SARS-CoV-2 genomes March–July 2020. **a**, Maximum-likelihood phylogenetic analysis of 391 viruses sampled from Bangladesh (black) on a background of 68,870 publicly available GISAID global sequences in collapsed clades. Collapsed clades are coloured by the predominant region/country that they were sampled in (blue, Europe; red, United States; green, India; pink, Middle East). International arrival frequencies from those regions between 18 February 2020 and 18 March 2020 are displayed in a bar chart and shown in the same colours. **b**, Maximum-likelihood Bayesian time-scaled phylogenetic analysis generated using BEAST (v.1.10.4); clades, internal nodes and migration events are annotated.

SARS-CoV-2-positive samples were collected by the IEDCR from patients in six administrative areas between March and July 2020 (Supplementary Table 1). Samples were tested for SARS-CoV-2 using RT–PCR and 67 positive samples were transferred to the Institute for Developing Science and Health Initiatives for sequencing. We combined our data with 324 publicly available genomes from Bangladesh that were sampled during the first wave (Supplementary Table 2, acknowledged in the Supplementary Information) and added 68,870 international genomes from GISAID for phylogeographical context (Fig. 1a).

The 391 isolates from Bangladesh fell into 19 lineages, assigned by the Pangolin lineage software^4^ (Pango lineages A, A.9, B, B.1, B.1.1, B.1.1.1, B.1.1.25, B.1.1.25.2, B.1.1.59, B.1.1.60, B.1.148, B.1.159, B.1.2, B.1.36, B.1.5, B.1.5.12, B.1.79, B.1.93 and B.2.1). Of the 19 lineages, 85% of isolates fell into the three dominant lineages all possessing single-nucleotide variants A23403G, C14408T and C3037T. The list of amino acid substitutions, insertions or deletions is provided separately in Supplementary Table 3. Bayesian phylodynamic analysis estimated that the mutation rate of the isolates from Bangladesh is 0.7 × 10^−3^ substitutions per site per year (~20 mutations per genome per year), which is consistent with global estimates^5^. Phylodynamic analysis of the most recent common ancestor (MRCA) predicted that SARS-CoV-2 first appeared in mid-February 2020 in Bangladesh (Fig. 1b), consistent with other global estimates^5^. The first positive case was detected in early March, within two weeks of the predicted first introduction.

### Population dynamics of SARS-CoV-2 in Bangladesh in 2020

**Fig. 2:**
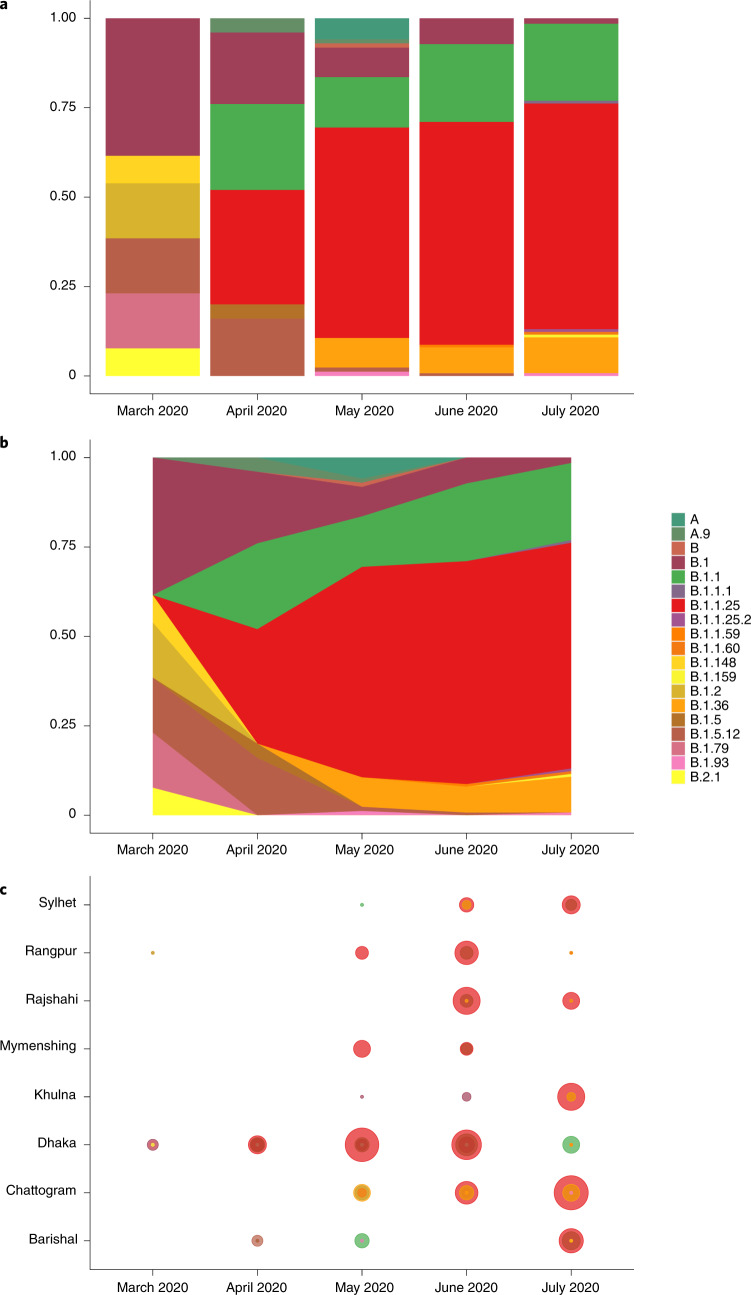
Lineage dynamics of 391 Bangladesh SARS-CoV-2 genomes March–July 2020. **a**, Proportional bar plot of Pango lineage diversity in Bangladesh between March and July 2020. **b**, Steam graph of the lineage dynamics between March and July 2020. **c**, Bubble plot of the representation of Pango lineages across eight divisions of Bangladesh.

On the basis of an analysis of the 391 sequences from Bangladesh that were sampled during the first wave, it is apparent that Pango lineage B.1 was the first SARS-CoV-2 lineage to be observed in Bangladesh, and had infected a traveller returning from Italy on 7 March. While B.1 predominated until the end of March, multiple other minor lineages were also introduced (B.1.5.12, B.2.1, B.1.2, B.1.148 and B.1.79) during March but failed to disseminate widely. After March, three lineages dominated—lineages B.1.1 and B.1.1.25 (first detected 5 April 2020 and 8 April 2020, respectively) and lineage B.1.36 (first detected at the end of March) (Fig. 2). None of these lineages shared an MRCA with other lineages present only in Bangladesh, rather, they were all derived from lineages that were established and circulating outside Bangladesh and that therefore represent separate introductions from the global outbreak pool (Fig. 1). B.1.1.25 has also been detected in the United Kingdom and Australia. Our data suggest that lineage B.1.1.25 was imported into Bangladesh at least twice before the cessation of international air travel on 21 March (Fig. 1a,b). B.1.1 seems to have been imported into Bangladesh at least five times, from both the United States and Europe (Fig. 1a). In contrast to lineages B.1.1 and B.1.1.25, phylogenetic analysis of lineage B.1.36 indicates that there was a single importation event linked to a traveller returning from Saudi Arabia who tested positive on 22 March in the Chattogram Division.

All three dominant lineages expanded clonally through sustained community transmission after the end of March (Fig. 2) to comprise 19% (B.1.1), 58% (B.1.1.25) and 8% (B.1.36) of all samples sequenced after this time. Lineages B.1.1 and B.1.1.25 were found dispersed throughout most divisions of Bangladesh (Fig. 2c). Lineage B.1.36 predominated in southern Bangladesh; 64% of isolates were found in the Chattogram Division. The remaining B.1.36 samples were found in Dhaka, Barisal, Khulna, Rajshahi, Rangpur and Sylhet, but only in small numbers (observed in up to two samples at each listed location).

Lineage A, which was present at the beginning of the pandemic in China in 2019 before the introduction into Europe in 2020, was not detected in Bangladesh until April 2020 (Fig. 1b), did not expand or disseminate widely, and may have been introduced by widespread dispersal in India before being detected in Bangladesh (Fig. 1a).

### Mass migration in Bangladesh at the end of March 2020

**Fig. 3:**
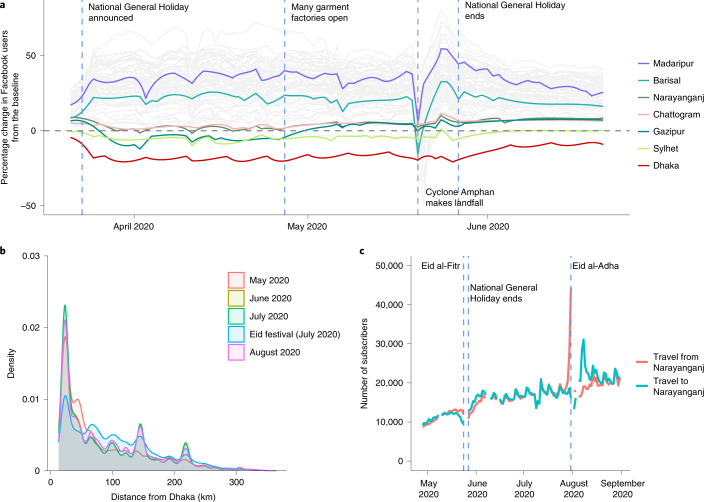
Population mobility dynamics in Bangladesh 2020. **a**, The percentage change in the Facebook user population over time for each district in Bangladesh (grey lines) compared with a baseline average. Specific districts are highlighted; the Dhaka district is shown in red. The dashed vertical lines indicate notable events. **b**, The distribution of trips from Dhaka by distance (km) travelled for each month and during Eid (29–31 July) based on call detail records data from three mobile phone operators. **c**, The number of subscribers travelling to and from Narayanganj.

Mobility data from Facebook users were available from 22 March, and from mobile phone operators from 27 April. An analysis of the mobility patterns among approximately 6,600 Facebook users (Fig. 3a) shows that around 14.2% of users left Dhaka between 23 and 26 March, indicating a mass migration out of Dhaka to all areas of the country. The displacement of individuals from the city to other parts of the country is illustrated by the increase in the population relative to the baseline in rural areas such as Barisal. Note the large spike of movement associated with the evacuation of Barisal due to Cyclone Amphan in May^13^. Interestingly, from Fig. 3a, in areas such as Gazipur and Narayanganj, it is apparent that, after initial displacement, people returned in late April^14^; our data show that this was followed by a return to Dhaka in late May (Fig. 3a).

Figure 3b illustrates the distribution of journey lengths, which are a proxy for movement patterns, taken by approximately 100 million mobile phone subscribers from three of four Bangladeshi operators from May to August. The impact of Eid—a national holiday during which many people travel to visit family and friends—is visible in July, with more long-distance trips occurring compared with other months. The percentage of long-distance trips (>50 km) travelled during the month of Eid (July) was significantly different compared with that of other months—the percentage of long-distance trips in July was 71.1% (95% confidence interval (CI) = 71.0–71.2%); the percentage of long-distance trips in August, the next highest month, was 57.9% (95% CI = 57.9–58.0%). In general, the distances travelled are considerable due to the country’s geography (Bangladesh spans 600 km east to west) and are consistent with Bangladesh’s highly mobile populations. Figure 3c shows the large number of subscribers travelling to and from Narayanganj, one of the first hotspots of SARS-CoV-2 and a continuing driver of transmission, throughout the summer. Together, these mobility data are consistent with the rapid dissemination of SARS-CoV-2 out of Dhaka to the rest of the country, as people left the city at the end of March, and frequent travel around Bangladesh sustaining transmission subsequently.

### Mass migration was the main driver of countrywide spread in 2020

Combining two data streams (population mobility and genomics data) revealed a link between the expansion of three dominant lineages and the mass migration that occurred at the end of March at the beginning of the general holiday. This is evidenced by the maximum clade credibility tree in Fig. 1b that dates the MRCA for all three dominant lineages directly before the observed mass migration when the mobility data usage swapped from cities to regional towns (Fig. 3). The resulting expansion of lineages B.1.1, B.1.1.25 and B.1.36 is shown in Fig. 2, which shows that these dominant lineages outcompeted other lineages, and reveals the rapid dissemination of these lineages to most areas of the country after the mass migration event at the end of March 2020.

Given the daily migration of the Bangladeshi population into and out of major cities, the risk for sustained transmission and expansion of SARS-CoV-2 remains extremely high and limits the effectiveness of behavioural interventions. This highly mobile population seems to have rapidly transported lineages across the country. This occurred mainly when workers living and working in cities returned home to rural areas because schools, offices and other working places closed.

### Genomic surveillance reveals Beta VOC dominance in 2021

To establish whether there had been recent changes in lineage dynamics or international importations, we sequenced a further 85 SARS-CoV-2 samples (Supplementary Table 4) in April 2021 that were sampled between November 2020 and April 2021. Of these 85 sequences, 30 were lineage B.1.1.25 (35%), 13 were VOC Alpha (B.1.1.7; 15%), 40 were VOC Beta (B.1.351; 47%), 1 was lineage B.1.1.315 and 1 was lineage B.1.525.

**Fig. 4:**
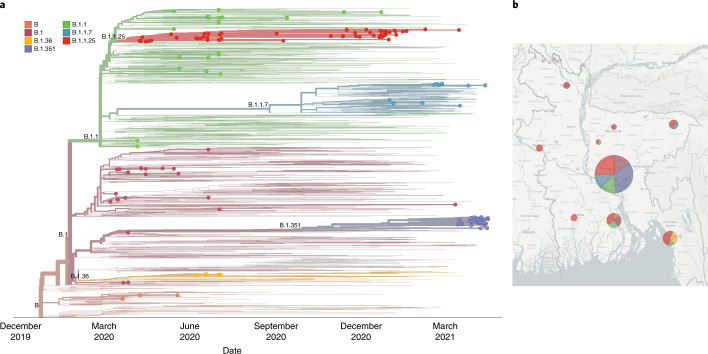
Bangladesh genomes in the context of a Nextstrain time-scaled global phylogeny. **a**, Nextstrain time-scaled phylogeny of 1,489 representative global sequences of SARS-CoV-2 sampled from GISAID on 20 April 2021. Pango lineages labelled on clades, and Bangladesh sequences (*n* = 166) are highlighted at the tips, coloured by lineage membership. **b**, Map of Bangladesh with pie chart representations of Pango lineage representation in each locality.

To investigate the emergence of lineages of concern Alpha (B.1.1.7) and Beta (B.1.351) in Bangladesh, a Nextstrain phylogenetic tree was built (Fig. 4). This revealed continuous transmission of lineage B.1.1.25 from the first wave and showed that B.1.1 and B.1.36 had been eliminated. Multiple introductions of Alpha B.1.1.7 occurred in Bangladesh after December 2020, but most were not established. More recently, in February 2021, a transmission chain became established in the community that resulted in non-travel-associated cases. Most concerning is the recent dominance of Beta (B.1.351), which has been assigned to a large number of cases that were not associated with travel and is responsible for 47% of our randomly sampled and recently sequenced cases. More than 85% of our Beta (B.1.351) sequences were sampled in Dhaka. Several other researchers who are sequencing viruses sampled in Bangladesh have deposited Beta (B.1.351) lineage sequences in GISAID (384 as of July 2021), indicating a lineage expansion and confirming that the dominance in our random sample is not a result of sampling bias. Further surveillance is necessary to investigate how this imported lineage has established and dominated the epidemic in Bangladesh so rapidly and what effect this lineage is having on transmissibility, mortality and immunity there.

## Discussion

In 2020, we sequenced 67 viral genomes from six administrative areas (divisions) of Bangladesh using a nanopore MinION device and combined these sequencing data with already-deposited global SARS-CoV-2 genomes (including 324 from Bangladesh COVID-19 cases) to show how early repeated international introductions into Bangladesh were replaced by endemic spread of three dominant lineages that dispersed the country in late March. Population mobility patterns analysed from digital trace and mobile phone data showed that the switch in the dynamics of SARS-CoV-2 genomic epidemiology coincided with migration out of cities to the rest of the country. In 2021, we again used nanopore sequencing to provide complete genome sequences of an additional 85 samples that were isolated between November 2020 and April 2021. The 2021 results revealed the presence of VOC Beta (B.1.351) in Bangladesh and its dominance in Dhaka, the capital of Bangladesh.

Our analyses of the genomic epidemiology of SARS-CoV-2 and population mobility in Bangladesh during the first wave of 2020 indicate that repeated international importations until late March were followed by a period of sustained community transmission that was consistent with a mass exodus from urban areas. A ban on international and domestic flights (21 March 2020) was probably the end point of international importations and 23 March marked the beginning of the expansion and dispersal of three dominant lineages in Bangladesh. The predicted introduction of SARS-CoV-2 into Bangladesh during mid-February is consistent with other global estimates that predicted that lineage B.1 was imported into Italy in late January but not detected until mid-February^4,5^.

Recent studies have linked a ~50 kb genomic locus inherited by modern humans from Neanderthals to a heightened risk of severe disease from COVID-19 (refs. ^15,16^). Importantly, the highest carrier frequency of this high-risk haplotype is found in Bangladesh, underlining the need for the continued strengthening of existing surveillance, monitoring systems and interventions to reduce transmission. To date, published studies reporting the effect of non-pharmaceutical interventions on lineage diversity have focused on HIC settings^17^. Although only a few studies have investigated the intricacies and specificities of differences in the dissemination of SARS-CoV-2 in low-income settings, those that have revealed that transmission dynamics can be completely different from HIC settings^18^.

Evidence from our study has revealed that stay-at-home orders can exacerbate transmission in LMICs with similar demographic specificities to Bangladesh by inducing mass migrations out of cities and return of transient workers to rural villages once work opportunities shut. Our finding, that non-pharmaceutical interventions (such as stay at home orders) that have been successful in HIC settings can’t always be applied identically in LMICs, is important and will continue to be relevant in light of the emergence and dominance of VOCs.

It is important to note that the Oxford–AstraZeneca vaccine was rolled out in Bangladesh from February 2021. The recent evidence of reduced efficacy of this vaccine against Beta (B.1.351) and Delta (B.1.617.2)^19^ further underscores the need for accurate and timely monitoring of VOCs. Moreover, the recent submission of other VOCs, including the Gamma variant (P.1) (February 2021) and Delta variant (B.1.617.2) (May 2021) in GISAID from Bangladesh illustrates the importance of real-time genomic surveillance to trace and reduce the possibility of countrywide spread. Our study exemplifies the advantages of combining mobility and genomic data to untangle outbreak dynamics to shape policies and interventions as the outbreak spreads. Here we provide evidence that policies that limit intracountry travel could reduce the incidence of VOCs in more rural areas of Bangladesh and should be swiftly implemented to prevent a rapid rise in cases outside of Dhaka.

Throughout the Bangladesh epidemic, our genomic epidemiology consortium has been in constant contact with key policy makers in Bangladesh. Since March 2020, we have been actively collaborating with national and international institutes on SARS-CoV-2 genomics and have provided genomic information to the Directorate General of Health Services (DGHS) and Ministry of Health and Family Welfare (MoFHW), Bangladesh. This has led to important policy decisions in response to the detection of the Alpha variant in early 2021 and Beta variant a few months later. For example, the government immediately quarantined patients who were positive for the SARS-CoV-2 Alpha variant (B.1.1.7), including travellers and their contacts, and enforced mandatory institutional quarantine for 14 days from 29 December 2020, for any incoming passengers from high-risk countries to Bangladesh.

Then, a few months later, when the Beta variant (B.1.351) emerged in Bangladesh, the government immediately quarantined infected individuals and their contacts, imposed restrictions on intercity movement and successfully limited the transmission across Bangladesh.

Moreover, in an important differentiation from interventions that were in place during the first wave, the government of Bangladesh also imposed a restriction on intercity movement (beginning June 2021) to prevent mass migration disseminating SARS-CoV-2 and VOCs across Bangladesh.

The global incidence of VOCs has continued to rise linearly throughout 2021, and lessons learnt during initial waves in 2020 (such as spread through mass migration) must be applied to stem the curve of the ongoing pandemic and increasingly transmissible variants^20^. Our study shows what can be achieved locally as a result of international collaboration using continuous genomic surveillance. Our consortium is committed to continuing to provide information on SARS-CoV-2 as the situation develops to give the required support to the Government of Bangladesh as well as to any other similar country settings.

We hope that our findings on outbreak dynamics in Bangladesh will help countries with similar healthcare challenges to develop research capabilities to monitor outbreaks and inform national social and healthcare policies to suppress the transmission of SARS-CoV-2.

## Methods

### Sample collection

As of 12 July 2021, the total number of COVID-19 testing facilities across Bangladesh was 613 (ref. ^3^). IEDCR was the first institute in the country to start testing for SARS-CoV-2 using RT–PCR. Thus, samples received at the IEDCR were available for sequencing from the start of the outbreak. In 2020, we sequenced 67 SARS-CoV-2-positive samples from between 8 March and 5 July. Furthermore, for our analysis, we included 324 SARS-CoV-2 strains from Bangladesh and 68,870 global SARS-CoV-2 sequences that had been deposited at the GISAID as of 31 July 2020, acknowledged in the Supplementary Information. In 2021, we sequenced an additional 85 SARS-CoV-2-positive samples that were obtained between 11 November 2020 and 15 April 2021.

### Whole-genome sequencing

Patient samples that were positive for SARS-CoV-2 on the basis of RT–PCR analysis were selected for sequencing. Stored nasopharyngeal swabs were re-extracted using the QIAamp Viral RNA Mini Kit (QIAGEN) and confirmed with RT–PCR using the WHO recommended primers and probes targeting the *E* and *N* genes. Samples with *C*_t_ values of less than 31 were retained for further analysis. The initial 67 samples were sequenced according to the Arctic nCoV-2019 sequencing protocol v2 (GunIt) (https://www.protocols.io/view/ncov-2019-sequencing-protocol-v2-bdp7i5rn?version_warning=no), while the later 85 samples were sequenced according to the Arctic nCoV-2019 sequencing protocol v3 (LoCost) (https://www.protocols.io/view/ncov-2019-sequencing-protocol-v3-locost-bh42j8ye). In brief, viral cDNA was synthesized using either SuperScript IV (Thermo Fisher Scientific) or the LunaScript RT SuperMix Kit (New England BioLabs) followed by second-strand synthesis using the Q5 high-fidelity DNA polymerase (New England BioLabs). Sequencing libraries were then constructed using the Oxford Nanopore ligation sequencing kit (SQK-LSK109). Libraries were sequenced using R9.4.1 MinION flow cells. Sequenced genomes were recovered by mapping pass quality reads against the reference Wuhan genome (GenBank: MN908947.3) using the ARTIC medaka (ARTIC-nCoV-bioinformaticsSOP-v.1.1.0) pipeline for sequence correction, single-nucleotide polymorphism calling and generation of the consensus sequence. We used high coverage (200×) to account for random sequencing errors, and regions with low coverage or low quality were masked using Ns. Lineages were assigned according to the proposed nomenclature of Pangolin lineage assignment software v.2.0.7 and lineage version 2020-08-29 (https://github.com/hCoV-2019/pangolin).

### Phylogenetic analysis

SARS-CoV-2 sequences from Bangladesh, deposited at the GISAID before 31 July 2020 (*n* = 391), were phylogenetically placed onto a global tree of 68,870 SARS-CoV-2 global sequences available from GISAID (31 July 2020). The total available (78,448) was filtered for those with short/ambiguous sequences to leave 68,870 sequences. The global tree was provided by R. Lanfear in his 31 July 2020 update^21^. The alignment of 68,870 sequences and the global tree was used with Llama software v.0.1 to place the 324 Bangladeshi sequenced strains in the global phylogenetic context^22^. Llama parameters, including a selection of five lineage representatives and an extraction of six from the larger tree radius, were used to investigate the phylogeography of the Bangladesh isolates.

We used TempEst v.1.5.3 to investigate the evolutionary tempo of the 2020 samples^23^ (Supplementary Fig. 1). We found a positive correlation with the temporal signal and a low to moderate association between genetic distances and sampling dates (*R*^2^ = 0.2471), reflecting the low mutation rate of SARS-CoV-2, which is consistent with findings reported elsewhere. Given the positive correlation, we determined that these data were suitable for phylogenetic molecular clock analysis in BEAST^24^. We used a time-aware coalescent Bayesian exponential growth model that is available in BEAST (v.1.10.4). The HKY+Γ model of nucleotide substitution was used with a strict molecular clock. Parameters were estimated using the Bayesian Markov Chain Monte Carlo framework, with 100,000,000-step-long chains, sampling every 1,000 steps and removing the initial 10% as burn-in. Sufficient sampling was assessed using Tracer (v.1.7.1), by verifying that every parameter had effective sampling sizes of more than 100. The resulting phylogenies were visualized with a maximum clade credibility tree in FigTree (v.1.4.4).

To update our phylogenetic analysis with our recent 2021 samples, we constructed a Nextstrain^25^ build using their global subsampling scheme across 1,045,491 sequences downloaded from GISAID on 20 April 20 2021. We selected one representative sequence from each country, year and month from the downloaded global sample. This resulted in 1,317 background global strains to 166 Bangladesh sequences, 152 provided by this study and 14 provided for background context and acknowledged in the Supplementary Information.

### Mobility analysis

Anonymized and aggregated daily population location data of Facebook users in Bangladesh were provided by Facebook Data for Good^26^. These data capture Facebook users who provide location information through the Facebook app by having location services enabled. The population of Facebook users is determined by the modal location for each individual during every eight-hour time window. The data analysed here were aggregated temporally (daily) and spatially (districts) and represent the daily count of Facebook users in each district from 22 March 2020 onwards. We compared these counts to the average number of users in each location during a 45-day baseline period preceding 22 March. The baseline averages were calculated for each unique day of the week and time of day combination. For each district, we calculated the daily percentage change in Facebook users compared with the corresponding baseline average.

We derived population mobility estimates from mobile phone call detail records provided by three of the four telecommunication operators (Grameenphone, Banglalink and Robi Axiata Limited) in Bangladesh. Trips were calculated based on changes in a subscriber’s assigned tower location from the previous day. All data were aggregated temporally (daily) and spatially (Upazilas; sub-district) on the basis of tower locations according to previously described methods^11,27,28^. The aggregated data consist of the daily number of subscribers for each Upazila and the total number of trips between all pairs of Upazilas from 27 April 2020 onwards. Although the Facebook data provide greater temporal coverage, they represent a much smaller percentage of the Bangladeshi population. The mobile phone data represent around 100 million subscribers across Bangladesh and enable us to estimate the daily number of trips each month, including the Eid holiday period, at the end of May 2020.

### Statistics and reproducibility

No statistical method was used to predetermine sample size and no data were excluded from the analyses unless *C*_t_ > 31.

### Reporting Summary

Further information on research design is available in the Nature Research Reporting Summary linked to this article.

## Supplementary information

Supplementary Information Supplementary Fig. 1 and Tables 1–4.

Reporting Summary

Supplementary Data 1 GISAID acknowledgements.

Peer Review Information

## Data Availability

All sequencing data used in this study are available on GISAID, as described in the Supplementary Information and in the GISAID acknowledgements (Supplementary Data 1). Sequencing reads generated in this study have been made available on the NCBI Sequence Read Archive service (BioProject ID: PRJNA737194).
