# Supplementary material for: Genomics, social media and mobile phone data enable mapping of SARS-CoV-2 lineages to inform health policy in Bangladesh
Source: Nat Microbiol. 2021 Sep 8;6(10):1271–8. doi: 10.1038/s41564-021-00955-3 (PMC8478645; doi:10.1038/s41564-021-00955-3)
Supplement: Supplementary file 1 — Supplementary Fig. 1 and Tables 1–4. [file 41564_2021_955_MOESM1_ESM.pdf]

nature portfolio

https://doi.org/10.1038/s41564-021-00955-3

Supplementary information

Genomics, social media and mobile phone data enable mapping of SARS-CoV-2 lineages to inform health policy in Bangladesh

In the format provided by the authors and unedited

Supplementary Material

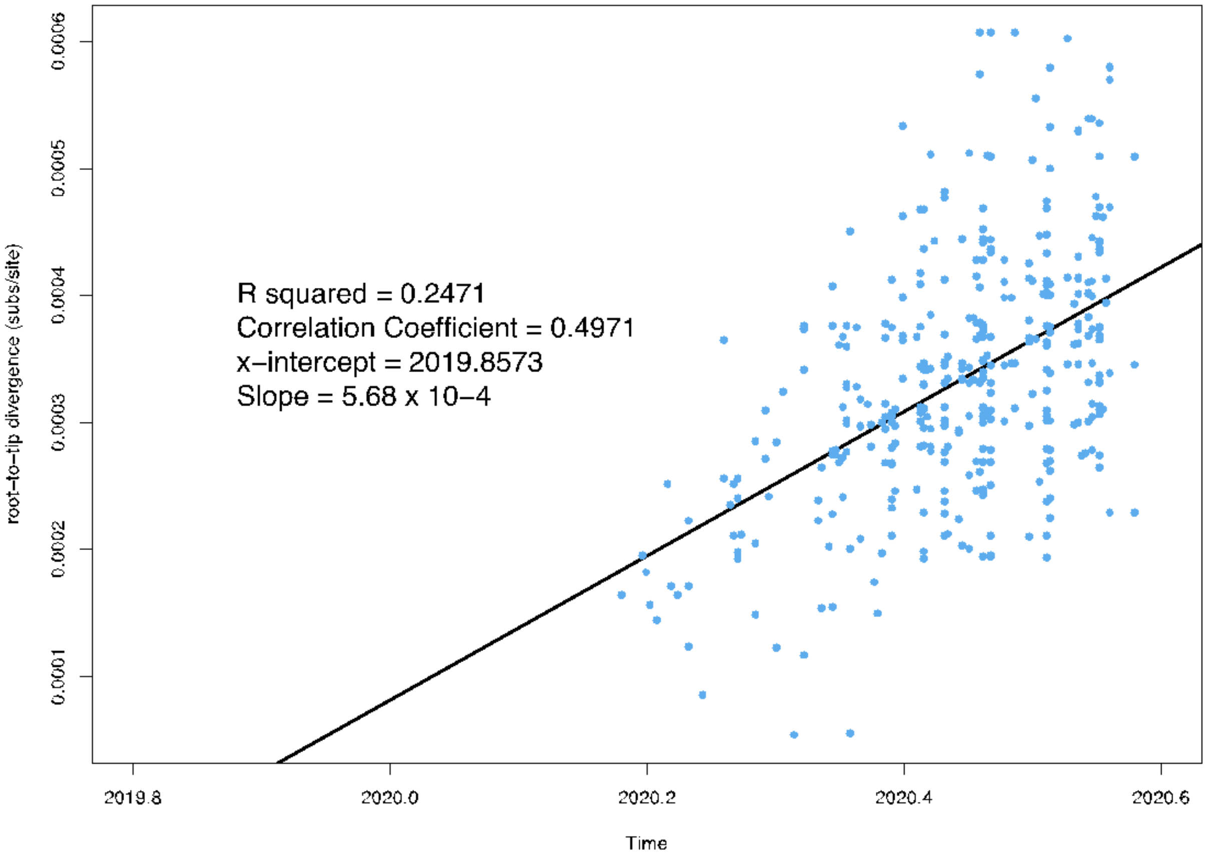

Supplementary Figure 1: Root-to-tip divergence plot for 391 sequences sampled in Bangladesh between March and July 2020. Evolutionary tempo was assessed using TempEst v.1.5 and data visualized in R.

Supplementary Table 1: 67 SARS-CoV-2 samples sequenced (Mar 2020 – Jul 2020) and metadata of sample date, Ct value, District, Sex, Age, Travel history, and Pangolin lineage. NA = Not available

Strain name	GISAID Accession	Date	Ct Value	Division	District, Area	Sex	Age (Year)	Travel History	Lineage
G-85	EPI_ISL_600570	May 14, 2020	17·6	Dhaka	Dhaka	Male	37	Contact of COVID-19 positive	B.1.1
G-66	EPI_ISL_600567	Apr 7, 2020	29·5	Dhaka	Narayanganj	NA	69	NA	B.1.1
G-40	EPI_ISL_600564	Mar 26, 2020	20·9	Dhaka	Dhaka	NA	NA	NA	B.1.2
G-38	EPI_ISL_600562	Mar 26, 2020	16·3	Dhaka	Dhaka	NA	55	Contact of USA returnee	B.1.79
G-31	EPI_ISL_600559	Mar 26, 2020	16·4	Dhaka	Dhaka	Female	NA	Italy returnee	B.1.79
G-29	EPI_ISL_600557	Mar 23, 2020	22·9	Dhaka	Dhaka	NA	NA	NA	B.1.148
G-27	EPI_ISL_600554	Mar 26, 2020	19·7	Rangpur	Gaibandha	NA	NA	Contact of Sample ID G-23	B.1
G-24	EPI_ISL_600552	Mar 14, 2020	28·2	Dhaka	Madaripur	NA	NA	Contact of Italy returnee	B.1.5.12
G-23	EPI_ISL_600549	Mar 21, 2020	21·5	Rangpur	Gaibandha	Female	48	USA Returnee	B.1.2
G-220	EPI_ISL_600546	May 20, 2020	23·1	Barishal	Barishal	NA	NA	NA	B.1.1
G-219	EPI_ISL_600543	May 14, 2020	20·2	Barishal	Barishal	NA	NA	NA	B.1.1
G-218	EPI_ISL_600541	May 20, 2020	28·1	Barishal	Barishal	NA	NA	NA	B.1.1
G-217	EPI_ISL_600538	May 17, 2020	24·6	Barishal	Barishal	NA	NA	NA	B.1.1
G-216	EPI_ISL_600535	May 11, 2020	23·0	Barishal	Barishal	NA	NA	NA	B.1.1
G-203	EPI_ISL_600533	May 19, 2020	22·0	Barishal	Barishal	NA	NA	NA	B.1.93
G-201	EPI_ISL_600530	May 18, 2020	24·4	Barishal	Barishal	NA	NA	NA	A.9
G-194	EPI_ISL_600527	Jul 5, 2020	21·6	Barishal	Barishal	NA	NA	NA	B.1.1
G-193	EPI_ISL_600524	Jul 3, 2020	16·4	Chattogram	Cox's Bazar	NA	NA	NA	B.1.36
G-192	EPI_ISL_600522	Jul 2, 2020	21·2	Chattogram	Cox's Bazar	NA	NA	NA	B.1.93
G-187	EPI_ISL_600519	Jun 15, 2020	26·0	Chattogram	Cox's Bazar	NA	NA	NA	B.1
G-185	EPI_ISL_600517	Jun 8, 2020	24·8	Chattogram	Cox's Bazar	NA	NA	NA	B.1.1
G-184	EPI_ISL_600515	Jun 7, 2020	23·1	Chattogram	Cox's Bazar	NA	NA	NA	B.1
G-183	EPI_ISL_600512	Jun 12, 2020	19·0	Chattogram	Cox's Bazar	NA	NA	NA	B.1.1
G-182	EPI_ISL_600510	Jun 16, 2020	19·7	Chattogram	Cox's Bazar	NA	NA	NA	B.1.1
G-176	EPI_ISL_600507	Jul 4, 2020	21·2	Barishal	Barishal	NA	NA	NA	B.1.1
G-173	EPI_ISL_600505	Jul 3, 2020	23·2	Chattogram	Cox's Bazar	NA	NA	NA	B.1.1.25.[illegible]
G-171	EPI_ISL_600502	Jul 1, 2020	18·3	Chattogram	Cox's Bazar	NA	NA	NA	B.1.36
G-168	EPI_ISL_600500	Jul 4, 2020	17·9	Chattogram	Cox's Bazar	NA	NA	NA	B.1.1
G-156	EPI_ISL_600498	Jun 18, 2020	22·0	Chattogram	Cox's Bazar	NA	NA	NA	B.1.36
G-152	EPI_ISL_600495	Jun 17, 2020	16·0	Chattogram	Cox's Bazar	NA	NA	NA	B.1.36
G-149	EPI_ISL_600492	Jun 8, 2020	18·1	Chattogram	Cox's Bazar	NA	NA	NA	B.1.36
G-144	EPI_ISL_600489	Jul 1, 2020	29·0	Dhaka	Dhaka	NA	NA	NA	B.1.1
G-142	EPI_ISL_600487	Jun 19, 2020	28·0	Dhaka	Dhaka	NA	NA	NA	B.1
G-14	EPI_ISL_600484	Apr 14, 2020	26·9	Dhaka	Narayanganj	NA	58	NA	B.1.1
G-134	EPI_ISL_600480	Jun 19, 2020	21·8	Dhaka	Dhaka	NA	NA	NA	B.1.1

G-131	EPI_ISL_600478	Jul 1, 2020	24.5	Dhaka	Dhaka	NA	NA	NA	B.1.1
G-130	EPI_ISL_600476	Jul 1, 2020	22·0	Dhaka	Dhaka	NA	NA	NA	B.1.1
G-129	EPI_ISL_600473	Jul 2, 2020	19·0	Dhaka	Dhaka	NA	NA	NA	B.1.1
G-127	EPI_ISL_600471	Jul 1, 2020	16·8	Dhaka	Dhaka	NA	NA	NA	B.1.1
G-126	EPI_ISL_600468	Jul 2, 2020	16·0	Dhaka	Dhaka	NA	NA	NA	B.1.1
G-123	EPI_ISL_600466	Jul 1, 2020	28·2	Dhaka	Dhaka	NA	NA	NA	B.1
G-119	EPI_ISL_600463	Jun 20, 2020	24·0	Dhaka	Dhaka	NA	NA	NA	B.1.5.12
G-115	EPI_ISL_600461	Jun 20, 2020	30·0	Dhaka	Dhaka	NA	NA	NA	B.1
G-111	EPI_ISL_600459	Jun 20, 2020	28·3	Dhaka	Dhaka	NA	NA	NA	B.1
G-110	EPI_ISL_600456	Jun 20, 2020	22·2	Dhaka	Dhaka	NA	NA	NA	B.1
G-11	EPI_ISL_600454	Apr 14, 2020	25·5	Dhaka	Dhaka	NA	NA	Contact of Italy returnee	B.1
G-108	EPI_ISL_600451	Jun 20, 2020	26·0	Dhaka	Dhaka	NA	NA	NA	B.1
G-106	EPI_ISL_600449	Jun 20, 2020	23·2	Dhaka	Dhaka	NA	NA	NA	B.1
G-104	EPI_ISL_600446	Jul 1, 2020	23·0	Dhaka	Dhaka	NA	NA	NA	B.1.1.1
G-103	EPI_ISL_600445	Jun 19, 2020	24·1	Dhaka	Dhaka	NA	NA	NA	B.1.1
G-10	EPI_ISL_600444	Apr 14, 2020	23·5	Dhaka	Narayanganj	NA	40	NA	B.1.1
638	EPI_ISL_600443	Apr 14, 2020	29·8	Dhaka	Dhaka	NA	NA	NA	B.1
374	EPI_ISL_600442	Mar 20, 2020	27·0	Dhaka	Dhaka	Male	32	NA	B.2.1
361	EPI_ISL_600441	Apr 5, 2020	26·3	Dhaka	Narayanganj	Female	38	NA	B.1.1
294	EPI_ISL_600440	Mar 17, 2020	23·0	Dhaka	Madaripur	Female	NA	Contact of Italy returnee	B.1
29156	EPI_ISL_600439	Apr 5, 2020	23·5	Dhaka	Dhaka	NA	NA	NA	B.1.1
2802	EPI_ISL_600438	Mar 7, 2020	29·7	Dhaka	Narayanganj	Male	NA	Italy returnee	B.1
2702	EPI_ISL_600437	May 2, 2020	19·6	Dhaka	Dhaka	Male	50	NA	B.1.5.12
256	EPI_ISL_600436	Mar 15, 2020	20·4	Dhaka	Dhaka	Male	74	NA	B.1
250	EPI_ISL_600435	Apr 10, 2020	26·2	Barishal	Barishal	Female	40	Contact of COVID-19 positive	B.1.5.12
203	EPI_ISL_600434	Apr 9, 2020	22·0	Barishal	Barishal	Male	6M	Contact of Sample ID 201	B.1.5
202	EPI_ISL_600433	Apr 9, 2020	24·2	Barishal	Barishal	Female	21	Contact of COVID-19 positive	B.1.5.12
201	EPI_ISL_600432	Apr 9, 2020	24·4	Barishal	Barishal	Male	27	Traveler	B.1.5.12
1757	EPI_ISL_600431	Mar 13, 2020	28·3	Dhaka	Dhaka	NA	NA	NA	B.1.5.12
1138	EPI_ISL_600430	Apr 20, 2020	27·2	Dhaka	Narayanganj	Male	45	NA	B.1.5.12
110	EPI_ISL_600429	Apr 8, 2020	31·5	Barishal	Barguna	Male	NA	NA	A.9
109879	EPI_ISL_600428	Apr 9, 2020	31·1	Barishal	Barishal	NA	35	NA	B.1.1

Supplementary Table 2: 324 SARS-CoV-2 sequences sampled in Bangladesh sourced from GISAID and metadata detailed on sample date, division collected in, assigned pangolin lineage

Strain name	Date	Division	Lineage	Originated
Bangladesh/NIB-BCSIR-02/2020	May 11, 2020	Dhaka	B	UK, China, USA
Bangladesh/NIB-01/2020	May 11, 2020	NA	B.1.1	UK, USA, Portugal
Bangladesh/NGRI-NSTU-31/2020	Jul 21, 2020	Chattogram	B.1.36	India, Saudi Arabia, Bangladesh
Bangladesh/NGRI-NSTU-21/2020	Jul 20, 2020	Chattogram	B.1.36	India, Saudi Arabia, Bangladesh
Bangladesh/NGRI-NSTU-18/2020	Jul 21, 2020	Chattogram	B.1.1.25	Bangladesh, UK, Australia
Bangladesh/NGRI-NSTU-16/2020	Jul 21, 2020	Chattogram	B.1.1.25	Bangladesh, UK, Australia
Bangladesh/NGRI-NSTU-06/2020	Jul 23, 2020	Chattogram	B.1	UK, USA, Australia
Bangladesh/NGRI-NSTU-05/2020	Jul 23, 2020	Chattogram	B.1.36	India, Saudi Arabia, Bangladesh
Bangladesh/NGRI-NSTU-03/2020	Jul 22, 2020	Chattogram	B.1.1.25	Bangladesh, UK, Australia
Bangladesh/NGRI-NSTU-02/2020	Jul 22, 2020	Chattogram	B.1.36	India, Saudi Arabia, Bangladesh
Bangladesh/JUST-GC40.86/2020	Jun 11, 2020	Khulna	B.1	UK, USA, Australia
Bangladesh/JUST-GC40.38/2020	Jun 11, 2020	Khulna	B.1	UK, USA, Australia
Bangladesh/JUST-GC15.09/2020	May 6, 2020	Khulna	B.1	UK, USA, Australia
Bangladesh/icddrb-2083/2020	Jun 7, 2020	NA	B.1.1.25	Bangladesh, UK, Australia
Bangladesh/DU-CARS-5586/2020	May 13, 2020	Dhaka	B.1.1.25	Bangladesh, UK, Australia
Bangladesh/DU-50768/2020	May 6, 2020	Dhaka	B.1	UK, USA, Australia
Bangladesh/DU-50761/2020	May 6, 2020	Dhaka	B.1.1.25	Bangladesh, UK, Australia
Bangladesh/DU-50758/2020	May 6, 2020	Dhaka	B.1.1.25	Bangladesh, UK, Australia
Bangladesh/DU-50740/2020	May 6, 2020	Dhaka	B.1.1.25	Bangladesh, UK, Australia
Bangladesh/DNAS-CPH-471/2020	Apr 28, 2020	Dhaka	B.1.1.25	Bangladesh, UK, Australia
Bangladesh/DNAS-CPH-467/2020	Apr 28, 2020	Dhaka	B.1.1.25	Bangladesh, UK, Australia
Bangladesh/DNAS-CPH-466/2020	Apr 28, 2020	Dhaka	B.1	UK, USA, Australia
Bangladesh/DNAS-CPH-436/2020	Apr 28, 2020	Dhaka	B.1.1.25	Bangladesh, UK, Australia
Bangladesh/DNAS-CPH-427/2020	Apr 28, 2020	Dhaka	B.1.1.25	Bangladesh, UK, Australia
Bangladesh/CHRF-0031/2020	Jun 16, 2020	NA	B.1.1.25	Bangladesh, UK, Australia
Bangladesh/CHRF-0030/2020	Jun 11, 2020	NA	B.1.36	India, Saudi Arabia, Bangladesh
Bangladesh/CHRF-0029/2020	Jun 17, 2020	NA	B.1.1.25	Bangladesh, UK, Australia
Bangladesh/CHRF-0028/2020	Jun 18, 2020	NA	B.1.1.25	Bangladesh, UK, Australia
Bangladesh/CHRF-0027/2020	Jun 16, 2020	NA	B.1.1.25	Bangladesh, UK, Australia
Bangladesh/CHRF-0025/2020	Jun 14, 2020	NA	B.1.1.25	Bangladesh, UK, Australia
Bangladesh/CHRF-0024/2020	Jun 14, 2020	NA	B.1.36	India, Saudi Arabia, Bangladesh
Bangladesh/CHRF-0023/2020	Jun 12, 2020	NA	B.1.1.25	Bangladesh, UK, Australia
Bangladesh/CHRF-0022/2020	Jun 14, 2020	NA	B.1.1.25	Bangladesh, UK, Australia
Bangladesh/CHRF-0021/2020	May 14, 2020	NA	B.1	UK, USA, Australia
Bangladesh/CHRF-0020/2020	May 30, 2020	NA	B.1.1.25	Bangladesh, UK, Australia
Bangladesh/CHRF-0019/2020	May 14, 2020	NA	B.1	UK, USA, Australia
Bangladesh/CHRF-0018/2020	May 8, 2020	NA	B.1.1.25	Bangladesh, UK, Australia
Bangladesh/CHRF-0017/2020	May 30, 2020	NA	B.1.1.25	Bangladesh, UK, Australia

Bangladesh/CHRF-0016/2020	May 23, 2020	NA	B.1.1.25	Bangladesh, UK, Australia
Bangladesh/CHRF-0015/2020	May 2, 2020	NA	B.1	UK, USA, Australia
Bangladesh/CHRF-0014/2020	May 21, 2020	NA	B.1.1.25	Bangladesh, UK, Australia
Bangladesh/CHRF-0013/2020	May 9, 2020	NA	B.1.1.25	Bangladesh, UK, Australia
Bangladesh/CHRF-0012/2020	May 5, 2020	NA	B.1.1.25	Bangladesh, UK, Australia
Bangladesh/CHRF-0011/2020	Mar 30, 2020	NA	B.1	UK, USA, Australia
Bangladesh/CHRF-0010/2020	May 3, 2020	Dhaka	B.1	UK, USA, Australia
Bangladesh/CHRF-0009/2020	Apr 8, 2020	Dhaka	B.1.1.25	Bangladesh, UK, Australia
Bangladesh/CHRF-0008/2020	May 16, 2020	Dhaka	B.1.1.25	Bangladesh, UK, Australia
Bangladesh/CHRF-0007/2020	May 14, 2020	Dhaka	B.1	UK, USA, Australia
Bangladesh/CHRF-0006/2020	May 17, 2020	Chattogram	B.1.36	India, Saudi Arabia, Bangladesh
Bangladesh/CHRF-0005/2020	May 10, 2020	Dhaka	B.1.1.25	Bangladesh, UK, Australia
Bangladesh/CHRF-0004/2020	Apr 17, 2020	Dhaka	B.1.1.25	Bangladesh, UK, Australia
Bangladesh/CHRF-0003/2020	Apr 17, 2020	Dhaka	B.1.1.25	Bangladesh, UK, Australia
Bangladesh/CHRF-0002/2020	Apr 20, 2020	Dhaka	B.1	UK, USA, Australia
Bangladesh/CHRF-0001/2020	Apr 18, 2020	Dhaka	B.1.1.25	Bangladesh, UK, Australia
Bangladesh/BCSIR-NILMRC-387/2020	Jul 18, 2020	Chattogram	B.1.1.25	Bangladesh, UK, Australia
Bangladesh/BCSIR-NILMRC-386/2020	Jul 21, 2020	Chattogram	B.1.1.25	Bangladesh, UK, Australia
Bangladesh/BCSIR-NILMRC-385/2020	Jul 21, 2020	Chattogram	B.1.1.25	Bangladesh, UK, Australia
Bangladesh/BCSIR-NILMRC-384/2020	Jul 21, 2020	Chattogram	B.1.1.25	Bangladesh, UK, Australia
Bangladesh/BCSIR-NILMRC-383/2020	Jul 21, 2020	Chattogram	B.1.1.25	Bangladesh, UK, Australia
Bangladesh/BCSIR-NILMRC-382/2020	Jul 21, 2020	Chattogram	B.1.1.25	Bangladesh, UK, Australia
Bangladesh/BCSIR-NILMRC-381/2020	Jul 21, 2020	Chattogram	B.1.36	India, Saudi Arabia, Bangladesh
Bangladesh/BCSIR-NILMRC-374/2020	Jul 31, 2020	Dhaka	B.1.1.60	UK, Austria, Bangladesh
Bangladesh/BCSIR-NILMRC-372/2020	Jul 31, 2020	Dhaka	B.1.1	UK, USA, Portugal
Bangladesh/BCSIR-NILMRC-371/2020	Jul 31, 2020	Dhaka	B.1.1.25	Bangladesh, UK, Australia
Bangladesh/BCSIR-NILMRC-370/2020	Jul 21, 2020	Chattogram	B.1.1.25	Bangladesh, UK, Australia
Bangladesh/BCSIR-NILMRC-369/2020	Jul 21, 2020	Chattogram	B.1.1.25	Bangladesh, UK, Australia
Bangladesh/BCSIR-NILMRC-368/2020	Jul 21, 2020	Chattogram	B.1.1.25	Bangladesh, UK, Australia
Bangladesh/BCSIR-NILMRC-367/2020	Jul 21, 2020	Chattogram	B.1.1.25	Bangladesh, UK, Australia
Bangladesh/BCSIR-NILMRC-365/2020	Jul 21, 2020	Chattogram	B.1.1	UK, USA, Portugal
Bangladesh/BCSIR-NILMRC-364/2020	Jul 24, 2020	Chattogram	B.1.1.25	Bangladesh, UK, Australia
Bangladesh/BCSIR-NILMRC-363/2020	Jul 24, 2020	Chattogram	B.1.1	UK, USA, Portugal
Bangladesh/BCSIR-NILMRC-362/2020	Jul 24, 2020	Chattogram	B.1.1	UK, USA, Portugal
Bangladesh/BCSIR-NILMRC-361/2020	Jul 24, 2020	Chattogram	B.1.1	UK, USA, Portugal
Bangladesh/BCSIR-NILMRC-359/2020	Jul 24, 2020	Chattogram	B.1.1.25	Bangladesh, UK, Australia
Bangladesh/BCSIR-NILMRC-358/2020	Jul 24, 2020	Chattogram	B.1.1.25	Bangladesh, UK, Australia
Bangladesh/BCSIR-NILMRC-356/2020	Jul 24, 2020	Chattogram	B.1.1.25	Bangladesh, UK, Australia
Bangladesh/BCSIR-NILMRC-354/2020	Jul 15, 2020	Khulna	B.1.1.25	Bangladesh, UK, Australia

Bangladesh/BCSIR-NILMRC-353/2020	Jul 15, 2020	Khulna	B.1.1.25	Bangladesh, UK, Australia
Bangladesh/BCSIR-NILMRC-352/2020	Jul 15, 2020	Khulna	B.1.1.25	Bangladesh, UK, Australia
Bangladesh/BCSIR-NILMRC-351/2020	Jul 15, 2020	Khulna	B.1.36	India, Saudi Arabia, Bangladesh
Bangladesh/BCSIR-NILMRC-350/2020	Jul 15, 2020	Khulna	B.1.1.25	Bangladesh, UK, Australia
Bangladesh/BCSIR-NILMRC-349/2020	Jul 15, 2020	Khulna	B.1.1.25	Bangladesh, UK, Australia
Bangladesh/BCSIR-NILMRC-346/2020	Jul 15, 2020	Rajshahi	B.1.1	UK, USA, Portugal
Bangladesh/BCSIR-NILMRC-345/2020	Jul 12, 2020	Rajshahi	B.1.1.25	Bangladesh, UK, Australia
Bangladesh/BCSIR-NILMRC-344/2020	Jul 12, 2020	Rajshahi	B.1.1.25	Bangladesh, UK, Australia
Bangladesh/BCSIR-NILMRC-343/2020	Jul 15, 2020	Rajshahi	B.1.36	India, Saudi Arabia, Bangladesh
Bangladesh/BCSIR-NILMRC-342/2020	Jul 15, 2020	Rajshahi	B.1.1.25	Bangladesh, UK, Australia
Bangladesh/BCSIR-NILMRC-341/2020	Jul 15, 2020	Rajshahi	B.1.1.25	Bangladesh, UK, Australia
Bangladesh/BCSIR-NILMRC-340/2020	Jul 15, 2020	Rajshahi	B.1.1.25	Bangladesh, UK, Australia
Bangladesh/BCSIR-NILMRC-339/2020	Jul 15, 2020	Rajshahi	B.1.1.25	Bangladesh, UK, Australia
Bangladesh/BCSIR-NILMRC-338/2020	Jul 15, 2020	Rajshahi	B.1.1.25	Bangladesh, UK, Australia
Bangladesh/BCSIR-NILMRC-333/2020	Jun 4, 2020	Mymensingh	B.1.1.25	Bangladesh, UK, Australia
Bangladesh/BCSIR-NILMRC-330/2020	Jun 3, 2020	Mymensingh	B.1.1.25	Bangladesh, UK, Australia
Bangladesh/BCSIR-NILMRC-329/2020	Jun 3, 2020	Mymensingh	B.1.1	UK, USA, Portugal
Bangladesh/BCSIR-NILMRC-328/2020	Jun 3, 2020	Mymensingh	B.1.1.25	Bangladesh, UK, Australia
Bangladesh/BCSIR-NILMRC-327/2020	Jun 3, 2020	Mymensingh	B.1.1	UK, USA, Portugal
Bangladesh/BCSIR-NILMRC-326/2020	Jun 3, 2020	Mymensingh	B.1.1	UK, USA, Portugal
Bangladesh/BCSIR-NILMRC-323/2020	Jun 3, 2020	Mymensingh	B.1.1.25	Bangladesh, UK, Australia
Bangladesh/BCSIR-NILMRC-322/2020	May 31, 2020	Mymensingh	B.1.1.25	Bangladesh, UK, Australia
Bangladesh/BCSIR-NILMRC-321/2020	May 31, 2020	Mymensingh	B.1.1.25	Bangladesh, UK, Australia
Bangladesh/BCSIR-NILMRC-320/2020	May 31, 2020	Mymensingh	B.1.1.25	Bangladesh, UK, Australia
Bangladesh/BCSIR-NILMRC-319/2020	May 31, 2020	Mymensingh	B.1.1.25	Bangladesh, UK, Australia
Bangladesh/BCSIR-NILMRC-318/2020	May 31, 2020	Mymensingh	B.1.1.25	Bangladesh, UK, Australia
Bangladesh/BCSIR-NILMRC-317/2020	May 31, 2020	Mymensingh	B.1.1.25	Bangladesh, UK, Australia
Bangladesh/BCSIR-NILMRC-315/2020	May 31, 2020	Mymensingh	B.1.1.25	Bangladesh, UK, Australia
Bangladesh/BCSIR-NILMRC-312/2020	Jul 20, 2020	Sylhet	B.1.1.25	Bangladesh, UK, Australia
Bangladesh/BCSIR-NILMRC-311/2020	Jul 19, 2020	Sylhet	B.1.1	UK, USA, Portugal
Bangladesh/BCSIR-NILMRC-307/2020	Jul 18, 2020	Sylhet	B.1.1.25	Bangladesh, UK, Australia
Bangladesh/BCSIR-NILMRC-306/2020	Jul 18, 2020	Sylhet	B.1.1	UK, USA, Portugal
Bangladesh/BCSIR-NILMRC-305/2020	Jul 18, 2020	Sylhet	B.1.1	UK, USA, Portugal
Bangladesh/BCSIR-NILMRC-298/2020	Jul 18, 2020	Sylhet	B.1.1.25	Bangladesh, UK, Australia
Bangladesh/BCSIR-NILMRC-297/2020	Jul 18, 2020	Sylhet	B.1.1.25	Bangladesh, UK, Australia
Bangladesh/BCSIR-NILMRC-296/2020	Jul 18, 2020	Sylhet	B.1.1.25	Bangladesh, UK, Australia
Bangladesh/BCSIR-NILMRC-295/2020	Jul 18, 2020	Sylhet	B.1.1.25	Bangladesh, UK, Australia
Bangladesh/BCSIR-NILMRC-293/2020	May 31, 2020	Sylhet	B.1.1	UK, USA, Portugal
Bangladesh/BCSIR-NILMRC-292/2020	Jul 17, 2020	Sylhet	B.1.1.25	Bangladesh, UK, Australia

Bangladesh/BCSIR-NILMRC-291/2020	Jul 18, 2020	Sylhet	B.1.1.25	Bangladesh, UK, Australia
Bangladesh/BCSIR-NILMRC-290/2020	Jul 14, 2020	Rangpur	B.1.1.25	Bangladesh, UK, Australia
Bangladesh/BCSIR-NILMRC-288/2020	Jul 14, 2020	Rangpur	B.1.36	India, Saudi Arabia, Bangladesh
Bangladesh/BCSIR-NILMRC-287/2020	Jul 14, 2020	Rangpur	B.1.1	UK, USA, Portugal
Bangladesh/BCSIR-NILMRC-285/2020	Jul 21, 2020	Chattogram	B.1.1.25	Bangladesh, UK, Australia
Bangladesh/BCSIR-NILMRC-284/2020	Jul 21, 2020	Chattogram	B.1.1.25	Bangladesh, UK, Australia
Bangladesh/BCSIR-NILMRC-283/2020	Jul 21, 2020	Chattogram	B.1.1.25	Bangladesh, UK, Australia
Bangladesh/BCSIR-NILMRC-282/2020	Jul 21, 2020	Chattogram	B.1.1.25	Bangladesh, UK, Australia
Bangladesh/BCSIR-NILMRC-281/2020	Jul 21, 2020	Chattogram	B.1.1.25	Bangladesh, UK, Australia
Bangladesh/BCSIR-NILMRC-280/2020	Jul 21, 2020	Chattogram	B.1.1.25	Bangladesh, UK, Australia
Bangladesh/BCSIR-NILMRC-278/2020	Jul 19, 2020	Chattogram	B.1.1.25	Bangladesh, UK, Australia
Bangladesh/BCSIR-NILMRC-276/2020	Jul 19, 2020	Chattogram	B.1.1.25	Bangladesh, UK, Australia
Bangladesh/BCSIR-NILMRC-275/2020	Jul 19, 2020	Chattogram	B.1.1.25	Bangladesh, UK, Australia
Bangladesh/BCSIR-NILMRC-274/2020	Jul 19, 2020	Chattogram	B.1.1.25	Bangladesh, UK, Australia
Bangladesh/BCSIR-NILMRC-273/2020	Jul 19, 2020	Chattogram	B.1.1	UK, USA, Portugal
Bangladesh/BCSIR-NILMRC-269/2020	Jul 19, 2020	Chattogram	B.1.1.25	Bangladesh, UK, Australia
Bangladesh/BCSIR-NILMRC-268/2020	Jul 19, 2020	Chattogram	B.1.1.25	Bangladesh, UK, Australia
Bangladesh/BCSIR-NILMRC-265/2020	Jul 19, 2020	Chattogram	B.1.1.25	Bangladesh, UK, Australia
Bangladesh/BCSIR-NILMRC-264/2020	Jul 16, 2020	Chattogram	B.1.1.25	Bangladesh, UK, Australia
Bangladesh/BCSIR-NILMRC-263/2020	Jul 7, 2020	Khulna	B.1.1	UK, USA, Portugal
Bangladesh/BCSIR-NILMRC-262/2020	Jul 7, 2020	Khulna	B.1.1	UK, USA, Portugal
Bangladesh/BCSIR-NILMRC-261/2020	Jul 7, 2020	Khulna	B.1.1.25	Bangladesh, UK, Australia
Bangladesh/BCSIR-NILMRC-260/2020	Jul 7, 2020	Khulna	B.1.1.25	Bangladesh, UK, Australia
Bangladesh/BCSIR-NILMRC-259/2020	Jul 7, 2020	Khulna	B.1.1.25	Bangladesh, UK, Australia
Bangladesh/BCSIR-NILMRC-258/2020	Jul 7, 2020	Khulna	B.1.1.25	Bangladesh, UK, Australia
Bangladesh/BCSIR-NILMRC-257/2020	Jul 7, 2020	Khulna	B.1.36	India, Saudi Arabia, Bangladesh
Bangladesh/BCSIR-NILMRC-256/2020	Jul 7, 2020	Khulna	B.1.1.25	Bangladesh, UK, Australia
Bangladesh/BCSIR-NILMRC-255/2020	Jul 7, 2020	Khulna	B.1.1.25	Bangladesh, UK, Australia
Bangladesh/BCSIR-NILMRC-254/2020	Jul 7, 2020	Khulna	B.1.1.25	Bangladesh, UK, Australia
Bangladesh/BCSIR-NILMRC-253/2020	Jul 7, 2020	Khulna	B.1.1.25	Bangladesh, UK, Australia
Bangladesh/BCSIR-NILMRC-251/2020	Jul 7, 2020	Khulna	B.1.1.25	Bangladesh, UK, Australia
Bangladesh/BCSIR-NILMRC-250/2020	Jul 7, 2020	Khulna	B.1.1.25	Bangladesh, UK, Australia
Bangladesh/BCSIR-NILMRC-249/2020	Jul 7, 2020	Khulna	B.1.1.25	Bangladesh, UK, Australia
Bangladesh/BCSIR-NILMRC-248/2020	Jul 7, 2020	Khulna	B.1.1.25	Bangladesh, UK, Australia
Bangladesh/BCSIR-NILMRC-247/2020	Jul 7, 2020	Khulna	B.1.1.25	Bangladesh, UK, Australia
Bangladesh/BCSIR-NILMRC-246/2020	Jul 7, 2020	Khulna	B.1.1.25	Bangladesh, UK, Australia
Bangladesh/BCSIR-NILMRC-245/2020	Jul 6, 2020	Barishal	B.1.1.25	Bangladesh, UK, Australia
Bangladesh/BCSIR-NILMRC-244/2020	Jul 6, 2020	Barishal	B.1.1.25	Bangladesh, UK, Australia
Bangladesh/BCSIR-NILMRC-243/2020	Jul 6, 2020	Barishal	B.1.1.25	Bangladesh, UK, Australia

Bangladesh/BCSIR-NILMRC-242/2020	Jul 6, 2020	Barishal	B.1.1.25	Bangladesh, UK, Australia
Bangladesh/BCSIR-NILMRC-241/2020	Jul 6, 2020	Barishal	B.1.1.25	Bangladesh, UK, Australia
Bangladesh/BCSIR-NILMRC-240/2020	Jul 6, 2020	Barishal	B.1.1.25	Bangladesh, UK, Australia
Bangladesh/BCSIR-NILMRC-239/2020	Jul 6, 2020	Barishal	B.1.1.25	Bangladesh, UK, Australia
Bangladesh/BCSIR-NILMRC-238/2020	Jul 6, 2020	Barishal	B.1.1.25	Bangladesh, UK, Australia
Bangladesh/BCSIR-NILMRC-237/2020	Jul 6, 2020	Barishal	B.1.1.25	Bangladesh, UK, Australia
Bangladesh/BCSIR-NILMRC-236/2020	Jul 6, 2020	Barishal	B.1.1	UK, USA, Portugal
Bangladesh/BCSIR-NILMRC-235/2020	Jul 6, 2020	Barishal	B.1.1.25	Bangladesh, UK, Australia
Bangladesh/BCSIR-NILMRC-234/2020	Jul 6, 2020	Barishal	B.1.1	UK, USA, Portugal
Bangladesh/BCSIR-NILMRC-233/2020	Jul 6, 2020	Barishal	B.1.1	UK, USA, Portugal
Bangladesh/BCSIR-NILMRC-232/2020	Jul 6, 2020	Barishal	B.1.36	India, Saudi Arabia, Bangladesh
Bangladesh/BCSIR-NILMRC-231/2020	Jul 6, 2020	Barishal	B.1.1.25	Bangladesh, UK, Australia
Bangladesh/BCSIR-NILMRC-230/2020	Jul 6, 2020	Barishal	B.1.1.25	Bangladesh, UK, Australia
Bangladesh/BCSIR-NILMRC-229/2020	Jul 6, 2020	Barishal	B.1.1.25	Bangladesh, UK, Australia
Bangladesh/BCSIR-NILMRC-228/2020	Jul 6, 2020	Barishal	B.1.159	France, Bangladesh
Bangladesh/BCSIR-NILMRC-227/2020	Jul 6, 2020	Barishal	B.1.1.25	Bangladesh, UK, Australia
Bangladesh/BCSIR-NILMRC-226/2020	Jul 6, 2020	Barishal	B.1.1.25	Bangladesh, UK, Australia
Bangladesh/BCSIR-NILMRC-224/2020	Jul 6, 2020	Barishal	B.1.1	UK, USA, Portugal
Bangladesh/BCSIR-NILMRC-223/2020	Jul 6, 2020	Barishal	B.1.1	UK, USA, Portugal
Bangladesh/BCSIR-NILMRC-222/2020	Jul 6, 2020	Barishal	B.1.1	UK, USA, Portugal
Bangladesh/BCSIR-NILMRC-220/2020	Jun 24, 2020	Sylhet	B.1.36	India, Saudi Arabia, Bangladesh
Bangladesh/BCSIR-NILMRC-219/2020	Jun 24, 2020	Sylhet	B.1.1.25	Bangladesh, UK, Australia
Bangladesh/BCSIR-NILMRC-218/2020	Jun 24, 2020	Sylhet	B.1.1.25	Bangladesh, UK, Australia
Bangladesh/BCSIR-NILMRC-217/2020	Jun 24, 2020	Sylhet	B.1.1.25	Bangladesh, UK, Australia
Bangladesh/BCSIR-NILMRC-216/2020	Jun 24, 2020	Sylhet	B.1.36	India, Saudi Arabia, Bangladesh
Bangladesh/BCSIR-NILMRC-213/2020	Jun 14, 2020	Sylhet	B.1.1	UK, USA, Portugal
Bangladesh/BCSIR-NILMRC-212/2020	Jun 14, 2020	Sylhet	B.1.1.25	Bangladesh, UK, Australia
Bangladesh/BCSIR-NILMRC-211/2020	Jun 14, 2020	Sylhet	B.1.1.25	Bangladesh, UK, Australia
Bangladesh/BCSIR-NILMRC-210/2020	Jun 14, 2020	Sylhet	B.1.1	UK, USA, Portugal
Bangladesh/BCSIR-NILMRC-208/2020	Jun 27, 2020	Chattogram	B.1.1.25	Bangladesh, UK, Australia
Bangladesh/BCSIR-NILMRC-207/2020	Jun 27, 2020	Chattogram	B.1.1.25	Bangladesh, UK, Australia
Bangladesh/BCSIR-NILMRC-206/2020	Jun 27, 2020	Chattogram	B.1.1.25	Bangladesh, UK, Australia
Bangladesh/BCSIR-NILMRC-205/2020	Jun 26, 2020	Chattogram	B.1.1.25	Bangladesh, UK, Australia
Bangladesh/BCSIR-NILMRC-204/2020	Jun 26, 2020	Chattogram	B.1.36	India, Saudi Arabia, Bangladesh
Bangladesh/BCSIR-NILMRC-203/2020	Jun 20, 2020	Chattogram	B.1.1.25	Bangladesh, UK, Australia
Bangladesh/BCSIR-NILMRC-201/2020	Jun 20, 2020	Chattogram	B.1.1.25	Bangladesh, UK, Australia
Bangladesh/BCSIR-NILMRC-199/2020	Jun 20, 2020	Chattogram	B.1.1.25	Bangladesh, UK, Australia
Bangladesh/BCSIR-NILMRC-198/2020	Jun 20, 2020	Chattogram	B.1.1	UK, USA, Portugal
Bangladesh/BCSIR-NILMRC-196/2020	Jun 20, 2020	Chattogram	B.1.1.25	Bangladesh, UK, Australia

Bangladesh/BCSIR-NILMRC-195/2020	Jun 20, 2020	Chattogram	B.1.1.25	Bangladesh, UK, Australia
Bangladesh/BCSIR-NILMRC-194/2020	Jun 20, 2020	Chattogram	B.1.1	UK, USA, Portugal
Bangladesh/BCSIR-NILMRC-193/2020	Jun 17, 2020	Chattogram	B.1.1.25	Bangladesh, UK, Australia
Bangladesh/BCSIR-NILMRC-192/2020	Jun 17, 2020	Chattogram	B.1.1.25	Bangladesh, UK, Australia
Bangladesh/BCSIR-NILMRC-190/2020	Jun 17, 2020	Chattogram	B.1.1.59	UK, Bangladesh
Bangladesh/BCSIR-NILMRC-188/2020	Jun 16, 2020	Chattogram	B.1.1.25	Bangladesh, UK, Australia
Bangladesh/BCSIR-NILMRC-187/2020	Jun 16, 2020	Chattogram	B.1.1.25	Bangladesh, UK, Australia
Bangladesh/BCSIR-NILMRC-186/2020	Jun 16, 2020	Chattogram	B.1.36	India, Saudi Arabia, Bangladesh
Bangladesh/BCSIR-NILMRC-184/2020	Jun 20, 2020	Rajshahi	B.1.1.25	Bangladesh, UK, Australia
Bangladesh/BCSIR-NILMRC-182/2020	Jun 20, 2020	Rajshahi	B.1.1	UK, USA, Portugal
Bangladesh/BCSIR-NILMRC-181/2020	Jun 20, 2020	Rajshahi	B.1.1.25	Bangladesh, UK, Australia
Bangladesh/BCSIR-NILMRC-180/2020	Jun 20, 2020	Rajshahi	B.1.1.25	Bangladesh, UK, Australia
Bangladesh/BCSIR-NILMRC-179/2020	Jun 15, 2020	Rajshahi	B.1.1.25	Bangladesh, UK, Australia
Bangladesh/BCSIR-NILMRC-178/2020	Jun 15, 2020	Rajshahi	B.1.1.25	Bangladesh, UK, Australia
Bangladesh/BCSIR-NILMRC-177/2020	Jun 12, 2020	Rajshahi	B.1.1.25	Bangladesh, UK, Australia
Bangladesh/BCSIR-NILMRC-176/2020	Jun 12, 2020	Rajshahi	B.1.1.25	Bangladesh, UK, Australia
Bangladesh/BCSIR-NILMRC-175/2020	Jun 12, 2020	Rajshahi	B.1.1.25	Bangladesh, UK, Australia
Bangladesh/BCSIR-NILMRC-174/2020	Jun 12, 2020	Rajshahi	B.1.1.25	Bangladesh, UK, Australia
Bangladesh/BCSIR-NILMRC-173/2020	Jun 18, 2020	Rajshahi	B.1.1.25	Bangladesh, UK, Australia
Bangladesh/BCSIR-NILMRC-172/2020	Jun 18, 2020	Rajshahi	B.1.1.25	Bangladesh, UK, Australia
Bangladesh/BCSIR-NILMRC-168/2020	Jun 18, 2020	Rajshahi	B.1.1.25	Bangladesh, UK, Australia
Bangladesh/BCSIR-NILMRC-167/2020	Jun 18, 2020	Rajshahi	B.1.1.25	Bangladesh, UK, Australia
Bangladesh/BCSIR-NILMRC-165/2020	Jun 18, 2020	Rajshahi	B.1.1	UK, USA, Portugal
Bangladesh/BCSIR-NILMRC-161/2020	Jun 18, 2020	Rajshahi	B.1.1.25	Bangladesh, UK, Australia
Bangladesh/BCSIR-NILMRC-160/2020	Jun 18, 2020	Rajshahi	B.1.1.25	Bangladesh, UK, Australia
Bangladesh/BCSIR-NILMRC-158/2020	Jun 18, 2020	Rajshahi	B.1.36	India, Saudi Arabia, Bangladesh
Bangladesh/BCSIR-NILMRC-156/2020	Jun 18, 2020	Rajshahi	B.1.1	UK, USA, Portugal
Bangladesh/BCSIR-NILMRC-155/2020	Jun 18, 2020	Rajshahi	B.1.1.25	Bangladesh, UK, Australia
Bangladesh/BCSIR-NILMRC-154/2020	Jun 18, 2020	Rajshahi	B.1.1	UK, USA, Portugal
Bangladesh/BCSIR-NILMRC-149/2020	Jun 18, 2020	Dhaka	B.1.1.25	Bangladesh, UK, Australia
Bangladesh/BCSIR-NILMRC-144/2020	Jun 18, 2020	Dhaka	B.1.1.25	Bangladesh, UK, Australia
Bangladesh/BCSIR-NILMRC-141/2020	Jun 18, 2020	Dhaka	B.1.1.25	Bangladesh, UK, Australia
Bangladesh/BCSIR-NILMRC-138/2020	Jun 18, 2020	Dhaka	B.1.1.25	Bangladesh, UK, Australia
Bangladesh/BCSIR-NILMRC-137/2020	Jun 18, 2020	Dhaka	B.1.1.25	Bangladesh, UK, Australia
Bangladesh/BCSIR-NILMRC-135/2020	Jun 18, 2020	Dhaka	B.1.1.25	Bangladesh, UK, Australia
Bangladesh/BCSIR-NILMRC-133/2020	Jun 18, 2020	Dhaka	B.1.1.25	Bangladesh, UK, Australia
Bangladesh/BCSIR-NILMRC-132/2020	Jun 18, 2020	Dhaka	B.1.1.25	Bangladesh, UK, Australia
Bangladesh/BCSIR-NILMRC-130/2020	Jun 17, 2020	Dhaka	B.1.1	UK, USA, Portugal
Bangladesh/BCSIR-NILMRC-126/2020	Jun 17, 2020	Rajshahi	B.1.1.25	Bangladesh, UK, Australia

Bangladesh/BCSIR-NILMRC-118a/2020	Jun 18, 2020	Dhaka	B.1.1	UK, USA, Portugal
Bangladesh/BCSIR-NILMRC-118/2020	Jun 18, 2020	Dhaka	B.1.1	UK, USA, Portugal
Bangladesh/BCSIR-NILMRC-117/2020	Jun 7, 2020	Rangpur	B.1.1	UK, USA, Portugal
Bangladesh/BCSIR-NILMRC-116/2020	Jun 7, 2020	Rangpur	B.1.1.25	Bangladesh, UK, Australia
Bangladesh/BCSIR-NILMRC-115/2020	Jun 7, 2020	Rangpur	B.1.1.25	Bangladesh, UK, Australia
Bangladesh/BCSIR-NILMRC-114/2020	Jun 7, 2020	Rangpur	B.1.1	UK, USA, Portugal
Bangladesh/BCSIR-NILMRC-113/2020	Jun 7, 2020	Rangpur	B.1.1.25	Bangladesh, UK, Australia
Bangladesh/BCSIR-NILMRC-112/2020	Jun 7, 2020	Rangpur	B.1.1	UK, USA, Portugal
Bangladesh/BCSIR-NILMRC-111/2020	Jun 7, 2020	Rangpur	B.1.1.25	Bangladesh, UK, Australia
Bangladesh/BCSIR-NILMRC-110/2020	Jun 7, 2020	Rangpur	B.1.1.25	Bangladesh, UK, Australia
Bangladesh/BCSIR-NILMRC-109/2020	Jun 7, 2020	Rangpur	B.1.1	UK, USA, Portugal
Bangladesh/BCSIR-NILMRC-108/2020	Jun 7, 2020	Rangpur	B.1.1.25	Bangladesh, UK, Australia
Bangladesh/BCSIR-NILMRC-107/2020	Jun 7, 2020	Rangpur	B.1.1.25	Bangladesh, UK, Australia
Bangladesh/BCSIR-NILMRC-105/2020	Jun 7, 2020	Rangpur	B.1.1.25	Bangladesh, UK, Australia
Bangladesh/BCSIR-NILMRC-104/2020	Jun 7, 2020	Rangpur	B.1.1.25	Bangladesh, UK, Australia
Bangladesh/BCSIR-NILMRC-103/2020	Jun 7, 2020	Rangpur	B.1.1.25	Bangladesh, UK, Australia
Bangladesh/BCSIR-NILMRC-102/2020	Jun 7, 2020	Rangpur	B.1.1.25	Bangladesh, UK, Australia
Bangladesh/BCSIR-NILMRC-101/2020	Jun 7, 2020	Rangpur	B.1.1.25	Bangladesh, UK, Australia
Bangladesh/BCSIR-NILMRC-100/2020	May 23, 2020	Rangpur	B.1.1.25	Bangladesh, UK, Australia
Bangladesh/BCSIR-NILMRC-096/2020	May 23, 2020	Rangpur	B.1.1.25	Bangladesh, UK, Australia
Bangladesh/BCSIR-NILMRC-095/2020	May 23, 2020	Rangpur	B.1.1.25	Bangladesh, UK, Australia
Bangladesh/BCSIR-NILMRC-094/2020	May 23, 2020	Rangpur	B.1.1.25	Bangladesh, UK, Australia
Bangladesh/BCSIR-NILMRC-091/2020	Jun 2, 2020	Rangpur	B.1.1.25	Bangladesh, UK, Australia
Bangladesh/BCSIR-NILMRC-090/2020	Jun 2, 2020	Rangpur	B.1.1.25	Bangladesh, UK, Australia
Bangladesh/BCSIR-NILMRC-088/2020	May 9, 2020	Dhaka	B.1.1.25	Bangladesh, UK, Australia
Bangladesh/BCSIR-NILMRC-087/2020	May 9, 2020	Dhaka	B.1.1.25	Bangladesh, UK, Australia
Bangladesh/BCSIR-NILMRC-086/2020	Jun 1, 2020	Dhaka	B.1.1	UK, USA, Portugal
Bangladesh/BCSIR-NILMRC-085/2020	Jun 1, 2020	Dhaka	B.1.1.25	Bangladesh, UK, Australia
Bangladesh/BCSIR-NILMRC-084/2020	Jun 1, 2020	Dhaka	B.1.1	UK, USA, Portugal
Bangladesh/BCSIR-NILMRC-083/2020	Jun 1, 2020	Dhaka	B.1.1.25	Bangladesh, UK, Australia
Bangladesh/BCSIR-NILMRC-082/2020	Jun 1, 2020	Dhaka	B.1.1.25	Bangladesh, UK, Australia
Bangladesh/BCSIR-NILMRC-081/2020	Jun 1, 2020	Dhaka	B.1.1.25	Bangladesh, UK, Australia
Bangladesh/BCSIR-NILMRC-080/2020	Jun 1, 2020	Dhaka	B.1.1	UK, USA, Portugal
Bangladesh/BCSIR-NILMRC-078/2020	Jun 1, 2020	Dhaka	B.1.1	UK, USA, Portugal
Bangladesh/BCSIR-NILMRC-077/2020	Jun 1, 2020	Dhaka	B.1.1.25	Bangladesh, UK, Australia
Bangladesh/BCSIR-NILMRC-076/2020	Jun 1, 2020	Dhaka	B.1.1.25	Bangladesh, UK, Australia
Bangladesh/BCSIR-NILMRC-075/2020	Jun 1, 2020	Dhaka	B.1.1.25	Bangladesh, UK, Australia
Bangladesh/BCSIR-NILMRC-074/2020	Jun 1, 2020	Dhaka	B.1.1.25	Bangladesh, UK, Australia
Bangladesh/BCSIR-NILMRC-073/2020	Jun 1, 2020	Dhaka	B.1.1.25	Bangladesh, UK, Australia

Bangladesh/BCSIR-NILMRC-072/2020	May 26, 2020	Chattogram	B.1.1.25	Bangladesh, UK, Australia
Bangladesh/BCSIR-NILMRC-071/2020	May 26, 2020	Chattogram	B.1.36	India, Saudi Arabia, Bangladesh
Bangladesh/BCSIR-NILMRC-070/2020	May 26, 2020	Chattogram	B.1.36	India, Saudi Arabia, Bangladesh
Bangladesh/BCSIR-NILMRC-069/2020	May 26, 2020	Chattogram	B.1.36	India, Saudi Arabia, Bangladesh
Bangladesh/BCSIR-NILMRC-067/2020	May 26, 2020	Chattogram	B.1.36	India, Saudi Arabia, Bangladesh
Bangladesh/BCSIR-NILMRC-066/2020	May 26, 2020	Chattogram	B.1.1.25	Bangladesh, UK, Australia
Bangladesh/BCSIR-NILMRC-064/2020	May 31, 2020	Dhaka	B.1.1.25	Bangladesh, UK, Australia
Bangladesh/BCSIR-NILMRC-062/2020	May 31, 2020	Dhaka	B.1.1.25	Bangladesh, UK, Australia
Bangladesh/BCSIR-NILMRC-060/2020	May 10, 2020	Dhaka	B.1.1.25	Bangladesh, UK, Australia
Bangladesh/BCSIR-NILMRC-059/2020	May 10, 2020	Dhaka	B.1.1.25	Bangladesh, UK, Australia
Bangladesh/BCSIR-NILMRC-058/2020	May 10, 2020	Dhaka	B.1.1.25	Bangladesh, UK, Australia
Bangladesh/BCSIR-NILMRC-057/2020	May 10, 2020	Dhaka	B.1.1	UK, USA, Portugal
Bangladesh/BCSIR-NILMRC-056/2020	May 10, 2020	Dhaka	B.1.1.25	Bangladesh, UK, Australia
Bangladesh/BCSIR-NILMRC-054/2020	Jun 1, 2020	Dhaka	B.1.1.25	Bangladesh, UK, Australia
Bangladesh/BCSIR-NILMRC-053/2020	May 7, 2020	Dhaka	B.1.1.25	Bangladesh, UK, Australia
Bangladesh/BCSIR-NILMRC-052/2020	Jun 1, 2020	Dhaka	B.1.1	UK, USA, Portugal
Bangladesh/BCSIR-NILMRC-051/2020	May 7, 2020	Dhaka	B.1.1.25	Bangladesh, UK, Australia
Bangladesh/BCSIR-NILMRC-050/2020	May 21, 2020	Dhaka	B.1.1.25	Bangladesh, UK, Australia
Bangladesh/BCSIR-NILMRC-042/2020	May 23, 2020	Dhaka	B.1.1.25	Bangladesh, UK, Australia
Bangladesh/BCSIR-NILMRC-025/2020	May 24, 2020	Dhaka	B.1.1	UK, USA, Portugal
Bangladesh/BCSIR-NILMRC-021/2020	May 24, 2020	Dhaka	B.1.1.25	Bangladesh, UK, Australia
Bangladesh/BCSIR-NILMRC-018/2020	May 24, 2020	Dhaka	B.1.1	UK, USA, Portugal
Bangladesh/BCSIR-NILMRC-017/2020	May 24, 2020	Dhaka	B.1.1.25	Bangladesh, UK, Australia
Bangladesh/BCSIR-NILMRC-015/2020	May 23, 2020	Dhaka	B.1.1.25	Bangladesh, UK, Australia
Bangladesh/BCSIR-NILMRC-009/2020	May 23, 2020	Dhaka	B.1.1.25	Bangladesh, UK, Australia
Bangladesh/BCSIR-NILMRC-008/2020	May 21, 2020	Dhaka	B.1.1.25	Bangladesh, UK, Australia
Bangladesh/BCSIR-NILMRC-007/2020	May 21, 2020	Dhaka	B.1.1	UK, USA, Portugal
Bangladesh/BCSIR-NILMRC-006/2020	May 21, 2020	Dhaka	B.1.1.25	Bangladesh, UK, Australia
Bangladesh/BCSIR-NILMRC-006.2/2020	May 23, 2020	Dhaka	B.1.1.25	Bangladesh, UK, Australia
Bangladesh/BCSIR-NILMRC-005/2020	May 23, 2020	Dhaka	B.1.1.25	Bangladesh, UK, Australia
Bangladesh/BCSIR-NILMRC-004-2/2020	May 21, 2020	Dhaka	B.1.1.25	Bangladesh, UK, Australia
Bangladesh/BCSIR-NILMRC-004/2020	May 23, 2020	Dhaka	B.1.1.25	Bangladesh, UK, Australia
Bangladesh/BCSIR-NILMRC-003/2020	May 23, 2020	Dhaka	B.1.1.25	Bangladesh, UK, Australia
Bangladesh/BCSIR-NILMRC-002/2020	May 23, 2020	Dhaka	B.1.1.25	Bangladesh, UK, Australia
Bangladesh/BCSIR-NILMRC_153/2020	Jun 18, 2020	Rajshahi	B.1.1.25	Bangladesh, UK, Australia
Bangladesh/BCSIR-NILMRC_152/2020	Jun 18, 2020	Dhaka	B.1.1.25	Bangladesh, UK, Australia
Bangladesh/BCSIR-NILMRC_151/2020	Jun 18, 2020	Dhaka	B.1.1	UK, USA, Portugal
Bangladesh/BCSIR-NILMRC_150/2020	Jun 18, 2020	Dhaka	B.1.1.25	Bangladesh, UK, Australia
Bangladesh/BCSIR-NILMRC_146/2020	Jun 18, 2020	Dhaka	B.1.1.25	Bangladesh, UK, Australia

Bangladesh/BCSIR-NILMRC_145/2020	Jun 18, 2020	Dhaka	B.1.1.25	Bangladesh, UK, Australia
Bangladesh/BCSIR-NILMRC_139/2020	Jun 18, 2020	Dhaka	B.1.1.25	Bangladesh, UK, Australia
Bangladesh/BCSIR-NILMRC_131/2020	Jun 18, 2020	Dhaka	B.1.1	UK, USA, Portugal
Bangladesh/BCSIR-NILMRC_125/2020	Jun 17, 2020	Rajshahi	B.1.1.25	Bangladesh, UK, Australia
Bangladesh/BCSIR-DU-16/2020	Jul 12, 2020	Dhaka	B.1.36	India, Saudi Arabia, Bangladesh
Bangladesh/BARJ-CVASU-CTG-518/2020	May 10, 2020	Chattogram	A	China, India, Japan
Bangladesh/BARJ-CVASU-CTG-517/2020	May 3, 2020	Chattogram	B.1.36	India, Saudi Arabia, Bangladesh
Bangladesh/BARJ-CVASU-CTG-511/2020	May 9, 2020	Chattogram	B.1.36	India, Saudi Arabia, Bangladesh
Bangladesh/BARJ-CVASU-CTG-506/2020	May 8, 2020	Chattogram	A	China, India, Japan
Bangladesh/BARJ-CVASU-CTG-503/2020	May 13, 2020	Chattogram	A	China, India, Japan
Bangladesh/BARJ-CVASU-CTG-502/2020	May 13, 2020	Chattogram	A	China, India, Japan
Bangladesh/BARJ-CVASU-CTG-501/2020	May 10, 2020	Chattogram	A	China, India, Japan
Bangladesh/Akbiomed-01/2020	Apr 25, 2020	NA	B.1	UK, USA, Australia

Supplementary Table 3: Amino acid substitution, insertion, or deletion present in 152 Bangladeshi SARS-CoV-2 sequenced isolates

Gene	Amino acid substitution, insertion, deletion	Isolates acquired mutation (%)
orf1ab	NSP12_P323L	127 (83.6)
	NSP6_G107del, NSP6_S106del, NSP6_F108del	54 (35.5) each
	NSP2_I120F	50 (32.9)
	NSP5_K90R	42 (27.6)
	NSP2_T85I	41 (27)
	NSP13_I151V	39 (25.7)
	NSP3_N1778S, NSP3_P1228L	38 (25) each
	NSP6_W31Y, NSP6_L33M, NSP6_F34V, NSP6_Q30E	29 (19.1) each
	NSP6_ins35VL	26 (17.1)
	NSP6_T29P	18 (11.8)
	NSP3_T183I, NSP3_A890D	14 (9.2) each
	NSP3_I1412T	13 (8.6)
	NSP6_T29P	12 (7.9)
	NSP12_P227L	11 (7.2)
	NSP6_ins70V, NSP6_C68S, NSP6_L67I	8 (5.3) each
	NSP1_R24C, NSP1_E57K, NSP12_N491K, NSP13_M274I, NSP15_R206S, NSP2_S430L	6 (3.9) each
	NSP3_K837N, NSP3_M494I, NSP6_F66C, NSP8_T93I	5 (3.3) each
	NSP1_E57K, NSP3_L1359A, NSP3_C1362V, NSP3_L1358I, NSP3_L1357F, NSP3_L1361I, NSP3_Q1360T, NSP3_T1363Y, NSP3_T1356Y, NSP6_K61C, NSP6_M57N, NSP6_H64stop, NSP6_N40K, NSP6_Y38F, NSP6_P44T, NSP6_S53V, NSP6_M47Y, NSP6_I49Y, NSP6_M52Y, NSP6_F42L, NSP6_A41C, NSP6_A51C, NSP6_I50Y, NSP6_F66C, NSP6_H62Q, NSP6_F36V, NSP6_K63T, NSP6_A46C, NSP6_A56C, NSP6_M58D, NSP6_A54C, NSP6_F59del	4 (2.6) each
S	Spike_D614G	111 (73)
	Spike_N501Y	43 (28.3)
	Spike_A701V	41 (27)
	Spike_K417N, Spike_D215G, Spike_D80A	37 (24.3) each
	Spike_A243del, Spike_L244del, Spike_L242del	35 (23) each
	Spike_L18F	34 (22.4)
	Spike_D614G	26 (17.1)
	Spike_E484K	22 (14.5)
	Spike_P681R	18 (11.8)
	Spike_T716I, Spike_A570D, Spike_D1118H, Spike_S982A	14 (9.2) each
	Spike_P681H, Spike_A67V	13 (8.6) each

	Spike_H69del, Spike_V70del	10 (6.6) each
	Spike_V445A	8 (5.3)
	Spike_Y144F, Spike_V143del	7 (4.6) each
	Spike_Q134N, Spike_C136V, Spike_N137M, Spike_V126L, Spike_E132N, Spike_I128L, Spike_D138I, Spike_V127L, Spike_P139H, Spike_L141W, Spike_V130S, Spike_C131V	6 (3.9) each
	Spike_Y144del	5 (3.3)
	Spike_Q677H, Spike_F157L	4 (2.6) each
**ORF3a**	ORF3a_Q57H	44 (28.9)
	ORF3a_S171L	43 (28.3)
	ORF3a_Q38E	5 (3.3)
	ORF3a_S171L	4 (2.6)
**E**	E_P71L	41 (26.9)
**M**	M_K15N	8 (5.3)
	M_A2V	5 (3.3)
**ORF8**	ORF8_Q27stop, ORF8_R52I	14 (9.2) each
**ORF9**	ORF9_Y73C	14 (9.2)
**N**	N_R203K, N_G204R	73 (48) each
	N_T205I	42 (27.6)
	N_D3L, N_S235F	14 (9.2) each
	N_S194L	7 (4.6)
**ORF10**	ORF10_K68stop	10 (6.6)
**ORF11**	ORF11_I121L	9 (5.9)
**ORF12**	ORF12_D35Y	9 (5.9)
**ORF13**	ORF13_P36T	5 (3.3)

Supplemental Table 4: 85 SARS-CoV-2 samples sequenced (Nov 2020 – Apr 2021) and metadata of sample date, Ct value, Division, District, Sex, Age, and Pangolin lineage. NA = Not Available

Strain name	GISAID Accession	Sample Date	Ct Value	Division	District	Sex	Age (Year)	Lineage
ideSHi-054	EPI_ISL_1498134	Dec 20, 2020	22.74	Sirajganj	Sirajganj	Male	29	B.1.1.25
ideSHi-043	EPI_ISL_1498152	Dec 21, 2020	24.62	Cumilla	Cumilla	Male	42	B.1.1.25
ideSHi-046	EPI_ISL_1498146	Dec 22, 2020	14.85	Barishal	Barishal	Female	50	B.1.1.25
ideSHi-045	EPI_ISL_1498142	Dec 31, 2020	22.58	Sylhet	Sylhet	Male	22	B.1.1.7
ideSHi-042	EPI_ISL_1599181	Dec 26, 2020	18.93	Chattogram	Chattogram	Male	19	B.1.1.25
ideSHi-041	EPI_ISL_1498147	Dec 21, 2020	18.75	Rangpur	Rangpur	Male	22	B.1.1.25
ideSHi-044	EPI_ISL_1599182	Dec 31, 2020	19.31	Sylhet	Sylhet	Male	33	B.1.1.25
ideSHi-047	EPI_ISL_1498133	Dec 22, 2020	21.19	Barishal	Barishal	Female	70	B.1.1.25
ideSHi-055	EPI_ISL_1498148	Dec 27, 2020	17.52	Khulna	Khulna	Male	90	B.1.1.25
ideSHi-048	EPI_ISL_1599183	Dec 22, 2020	18.39	Barishal	Barishal	Male	32	B.1.1.25
ideSHi-052	EPI_ISL_1599184	Dec 23, 2020	19.12	Sirajganj	Rajshahi	Male	27	B.1.1.25
ideSHi-049	EPI_ISL_1498130	Dec 30, 2020	15.8	Sirajganj	Mymensingh	Female	63	B.1.1.25
ideSHi-057	EPI_ISL_1498129	Dec 27, 2020	21.85	Khulna	Khulna	Male	65	B.1.1.25
ideSHi-058	EPI_ISL_1498144	Dec 27, 2020	14.67	Khulna	Khulna	Male	68	B.1.1.25
ideSHi-059	EPI_ISL_1599185	Dec 25, 2020	12.51	NA	NA	Male	48	B.1.1.25
ideSHi-063	EPI_ISL_1599186	Jan 5, 2021	11.12	NA	Rajshahi	Female	43	B.1.1.25
ideSHi-034	EPI_ISL_1599187	Dec 10, 2020	23.99	Dhaka	Dhaka	Female	29	B.1.1.25
ideSHi-026	EPI_ISL_1498143	Jan 7, 2021	21.92	Dhaka	Dhaka	Male	32	B.1.1.25
ideSHi-025	EPI_ISL_1498135	Jan 18, 2021	24.6	Dhaka	Dhaka	Male	18	B.1.1.25
ideSHi-017	EPI_ISL_1498128	Dec 26, 2020	24.51	Dhaka	Dhaka	Male	28	B.1.1.25
ideSHi-057	EPI_ISL_1498126	Mar 15, 2021	20.17	NA	NA	Male	65	B.1.351
ideSHi-0210	EPI_ISL_1498137	Mar 15, 2021	18.34	NA	Dhaka	Male	57	B.1.351
ideSHi-0216	EPI_ISL_1498131	Mar 15, 2021	24.41	Dhaka	Dhaka	Male	55	B.1.1.25
ideSHi-078	EPI_ISL_1498136	Mar 16, 2021	20.5	Dhaka	Dhaka	Male	37	B.1.351
ideSHi-T-1784	EPI_ISL_1498145	Mar 11, 2021	20.07	Sunamganj	Sylhet	Male	24	B.1.525
ideSHi-N-1390	EPI_ISL_1498127	Mar 14, 2021	17.14	Dhaka	Dhaka	Male	61	B.1.351
ideSHi-N-1392	EPI_ISL_1498132	Mar 16, 2021	18.92	Dhaka	Dhaka	Male	59	B.1.1.7
ideSHi-N-1406	EPI_ISL_1498138	Mar 18, 2021	16.64	Dhaka	Dhaka	Male	51	B.1.351
ideSHi-N-1465	EPI_ISL_1498151	Mar 28, 2021	19.69	Dhaka	Dhaka	Female	26	B.1.351
ideSHi-T-3089	EPI_ISL_1498150	Mar 28, 2021	20.02	Mymensingh	Mymensingh	Male	50	B.1.351
ideSHi-N-1444	EPI_ISL_1498139	Mar 28, 2021	18.22	Dhaka	Dhaka	Male	74	B.1.351
ideSHi-N-1483	EPI_ISL_1498140	Mar 29, 2021	14.48	Dhaka	Dhaka	Male	52	B.1.351
ideSHi-N-1506	EPI_ISL_1498141	Mar 31, 2021	17.52	Dhaka	Dhaka	Male	29	B.1.351
ideSHi-N-1500	EPI_ISL_1498149	Mar 31, 2021	17.62	Dhaka	Dhaka	Female	32	B.1.351

ideSHi-B-100402007	EPI_ISL_1582390	Feb 4, 2021	19.16	Dhaka	Dhaka	Male	33	B.1.1.7
ideSHi-B-301702002	EPI_ISL_1582391	Feb 16, 2021	12.13	Dhaka	Dhaka	Male	55	B.1.1.7
ideSHi-IEDCR-233472	EPI_ISL_1582392	Feb 18, 2021	19.14	Dhaka	Dhaka	Female	23	B.1.1.7
ideSHi-IEDCR-233622	EPI_ISL_1582393	Feb 20, 2021	22.89	Dhaka	Dhaka	Female	53	B.1.1.7
ideSHi-B-302102001	EPI_ISL_1582394	Feb 21, 2021	17.82	Dhaka	Dhaka	Male	55	B.1.1.7
ideSHi-B-302302003	EPI_ISL_1582395	Feb 23, 2021	23.62	Dhaka	Dhaka	Male	22	B.1.1.7
ideSHi-B-302502004	EPI_ISL_1582396	Feb 25, 2021	18.24	Dhaka	Dhaka	Male	24	B.1.1.7
ideSHi-B-302502005	EPI_ISL_1582397	Feb 25, 2021	25.46	Dhaka	Dhaka	Female	53	B.1.1.7
ideSHi-ESP001	EPI_ISL_1575127	Apr 7, 2021	14.6	Dhaka	Dhaka	Male	65	B.1.351
ideSHi-ESP002	EPI_ISL_1587429	Mar 18, 2021	16.86	Dhaka	Dhaka	Female	56	B.1.351
ideSHi-ESP003	EPI_ISL_1593853	Mar 25, 2021	19	Dhaka	Dhaka	Male	47	B.1.1.7
ideSHi-IR-09	EPI_ISL_1587430	Apr 1, 2021	23.49	Dhaka	Dhaka	Female	52	B.1.351
ideSHi-IR-33	EPI_ISL_1587431	Apr 3, 2021	19.62	Dhaka	Dhaka	Male	50	B.1.351
ideSHi-IR-34	EPI_ISL_1587432	Apr 3, 2021	24.28	Dhaka	Dhaka	Male	38	B.1.351
ideSHi-IR-71	EPI_ISL_1587433	Apr 4, 2021	20.44	Dhaka	Dhaka	Male	34	B.1.351
ideSHi-TR-341	EPI_ISL_1593854	Apr 4, 2021	20.07	Habiganj	Sylhet	Male	36	B.1.351
ideSHi-IR-51	EPI_ISL_1587434	Apr 4, 2021	23.28	Dhaka	Dhaka	Male	42	B.1.351
ideSHi-IR-87	EPI_ISL_1587435	Apr 5, 2021	23.76	NA	NA	Female	45	B.1.351
ideSHi-IR-73	EPI_ISL_1587436	Apr 5, 2021	21.81	Dhaka	Dhaka	Male	38	B.1.351
ideSHi-IR-98	EPI_ISL_1587437	Apr 6, 2021	19.07	Dhaka	Dhaka	Female	71	B.1.351
ideSHi-IR-121	EPI_ISL_1587438	Apr 7, 2021	19.05	Dhaka	Dhaka	Female	43	B.1.351
ideSHi-IR-123	EPI_ISL_1587439	Apr 7, 2021	24.72	Dhaka	Dhaka	Male	54	B.1.351
ideSHi-TR-1069	EPI_ISL_1587440	Apr 8, 2021	21.48	Dhaka	Dhaka	Female	51	B.1.351
ideSHi-IR-137	EPI_ISL_1587441	Apr 8, 2021	19.37	Dhaka	Dhaka	Female	51	B.1.351
ideSHi-IR-179	EPI_ISL_1587442	Apr 8, 2021	18.46	Dhaka	Dhaka	Male	48	B.1.351
ideSHi-TR-1310	EPI_ISL_1587443	Apr 11, 2021	22.35	Noakhali	Chattogram	Male	42	B.1.351
ideSHi-TR-1381	EPI_ISL_1587444	Apr 11, 2021	23	Pirojpur	Barishal	Male	28	B.1.351
ideSHi-IR-182	EPI_ISL_1587445	Apr 11, 2021	21.02	Dhaka	Dhaka	Male	60	B.1.351
ideSHi-IR-197	EPI_ISL_1593855	Apr 11, 2021	25.8	Dhaka	Dhaka	Male	61	B.1.351
ideSHi-IR-172	EPI_ISL_1587446	Apr 10, 2021	19.76	Dhaka	Dhaka	Female	40	B.1.351
ideSHi-IR-1214	EPI_ISL_1587447	Apr 10, 2021	20.7	Dhaka	Dhaka	Male	29	B.1.351
ideSHi-KH007	EPI_ISL_1657075	Nov 12, 2020	25	Dhaka	Dhaka	Male	30	B.1.1.25
ideSHi-CP022	EPI_ISL_1657076	Jan 6, 2021	21.89	Dhaka	Dhaka	Female	35	B.1.1.25
ideSHi-MH029	EPI_ISL_1657077	Jan 14, 2021	17.57	Dhaka	Dhaka	Male	32	B.1.1.25
ideSHi-MH004	EPI_ISL_1657078	Nov 11, 2020	19.21	Dhaka	Dhaka	Female	37	B.1.1.315
ideSHi-CP012	EPI_ISL_1657079	Dec 6, 2020	22.04	Dhaka	Dhaka	Male	29	B.1.1.25
ideSHi-MH003	EPI_ISL_1657080	Nov 11, 2020	24.42	Dhaka	Dhaka	Female	37	B.1.1.25
ideSHi-KH015	EPI_ISL_1657081	Nov 29, 2020	20.64	Dhaka	Dhaka	Male	67	B.1.1.25
ideSHi-CP005	EPI_ISL_1669902	Nov 15, 2020	23.28	Dhaka	Dhaka	Male	53	B.1.1.25

ideSHi-MH007-D01	EPI_ISL_1657082	Nov 15, 2020	23.32	Dhaka	Dhaka	Male	45	B.1.1.25
ideSHi-CP030	EPI_ISL_1669903	Feb 7, 2021	22.75	Dhaka	Dhaka	Female	23	B.1.1.25
ideSHi-KH023	EPI_ISL_1657083	Dec 14, 2020	23.38	Dhaka	Dhaka	Male	50	B.1.1.25
ideSHi-IR-204	EPI_ISL_1669904	Apr 12, 2021	23.98	Dhaka	Dhaka	Female	27	B.1.1.7
ideSHi-IR-205	EPI_ISL_1669905	Apr 12, 2021	23.52	Dhaka	Dhaka	Male	31	B.1.1.7
ideSHi-IR-209	EPI_ISL_1657084	Apr 12, 2021	21.95	Dhaka	Dhaka	Male	74	B.1.351
ideSHi-IR-211	EPI_ISL_1669906	Apr 13, 2021	24.11	Dhaka	Dhaka	Male	33	B.1.351
ideSHi-IR-212	EPI_ISL_1657085	Apr 13, 2021	22.4	Dhaka	Dhaka	Male	69	B.1.351
ideSHi-IR-238	EPI_ISL_1657086	Apr 14, 2021	22.62	Dhaka	Dhaka	Female	60	B.1.351
ideSHi-IR-255	EPI_ISL_1669907	Apr 15, 2021	22.39	Dhaka	Dhaka	Female	35	B.1.351
ideSHi-IR-256	EPI_ISL_1657087	Apr 15, 2021	21.48	Dhaka	Dhaka	Female	30	B.1.351
ideSHi-IR-244	EPI_ISL_1657088	Apr 15, 2021	24.23	Dhaka	Dhaka	Male	46	B.1.351

GISAID acknowledgements
We gratefully acknowledge the Authors from the Originating laboratories responsible for obtaining the specimens, as well as the Submitting laboratories where the genome data were generated and shared via GISAID, on which this research is based. All acknowledgements for the global phylogeny produced by Rob Lanfear of all GISAID submissions up to 31/7/20 can be found at https://github.com/roblanf/sarscov2phylo/tree/31-7-20/acknowledgements

Bangladeshi strain GISAID acknowledgements can be found in additional file Bangladesh_gisaid_acknowledgement.pdf.
