# Supplementary material for: Genomics, social media and mobile phone data enable mapping of SARS-CoV-2 lineages to inform health policy in Bangladesh
Source: Nat Microbiol. 2021 Sep 8;6(10):1271–8. doi: 10.1038/s41564-021-00955-3 (PMC8478645; doi:10.1038/s41564-021-00955-3)
Supplement: Supplementary file 2 — Reporting Summary [file 41564_2021_955_MOESM2_ESM.pdf]

nature portfolio

Corresponding author(s): Firdausi Qadri

Last updated by author(s): Jul 23, 2021

Reporting Summary

Nature Portfolio wishes to improve the reproducibility of the work that we publish. This form provides structure for consistency and transparency in reporting. For further information on Nature Portfolio policies, see our Editorial Policies and the Editorial Policy Checklist.

Statistics

For all statistical analyses, confirm that the following items are present in the figure legend, table legend, main text, or Methods section.

n/a	Confirmed	
☐	☒	The exact sample size (*n*) for each experimental group/condition, given as a discrete number and unit of measurement
☐	☒	A statement on whether measurements were taken from distinct samples or whether the same sample was measured repeatedly
☐	☒	The statistical test(s) used AND whether they are one- or two-sided<br>*Only common tests should be described solely by name; describe more complex techniques in the Methods section.*
☒	☐	A description of all covariates tested
☐	☒	A description of any assumptions or corrections, such as tests of normality and adjustment for multiple comparisons
☐	☒	A full description of the statistical parameters including central tendency (e.g. means) or other basic estimates (e.g. regression coefficient) AND variation (e.g. standard deviation) or associated estimates of uncertainty (e.g. confidence intervals)
☐	☒	For null hypothesis testing, the test statistic (e.g. *F*, *t*, *r*) with confidence intervals, effect sizes, degrees of freedom and *P* value noted<br>*Give P values as exact values whenever suitable.*
☐	☒	For Bayesian analysis, information on the choice of priors and Markov chain Monte Carlo settings
☒	☐	For hierarchical and complex designs, identification of the appropriate level for tests and full reporting of outcomes
☒	☐	Estimates of effect sizes (e.g. Cohen's *d*, Pearson's *r*), indicating how they were calculated

Our web collection on statistics for biologists contains articles on many of the points above.

Software and code

Policy information about availability of computer code

Data collection	Sequencing data was collected through Oxford Nanopore software.
Data analysis	ARTIC medaka pipeline v1.1.0, pangolin lineage assignment software v2.0.7, llama software version 0.1, TempEst v.1.5.3, BEAST v 1.10.4, Tracer v 1.7.1, FigTree, v1.4.4,

For manuscripts utilizing custom algorithms or software that are central to the research but not yet described in published literature, software must be made available to editors and reviewers. We strongly encourage code deposition in a community repository (e.g. GitHub). See the Nature Portfolio guidelines for submitting code & software for further information.

Data

Policy information about availability of data

All manuscripts must include a data availability statement. This statement should provide the following information, where applicable:
- Accession codes, unique identifiers, or web links for publicly available datasets
- A description of any restrictions on data availability
- For clinical datasets or third party data, please ensure that the statement adheres to our policy

Data availability:
All consensus genomes are available from the GISAID database and sequencing reads from the NCBI Sequence Read Archive service (BioProject ID: PRJNA737194).

Field-specific reporting

Please select the one below that is the best fit for your research. If you are not sure, read the appropriate sections before making your selection.

☐ Life sciences ☐ Behavioural & social sciences ☒ Ecological, evolutionary & environmental sciences

For a reference copy of the document with all sections, see nature.com/documents/nr-reporting-summary-flat.pdf

Ecological, evolutionary & environmental sciences study design

All studies must disclose on these points even when the disclosure is negative.

Study description	In Bangladesh genomics, social media and mobile phone data streams are integrated to map the spread of SARS-CoV-2 lineages and inform country-level policies to curb infection rates
Research sample	We provide 152 whole genome sequences of SARS-CoV-2 sampled in Bangladesh between March 2020 and April 2021. All sequences are indepent and sampled from a clinical case of COVID-19.
Sampling strategy	No statistical method was used to predetermine sample size and no data were excluded from the analyses unless ct value <31.
Data collection	All data was collected in Bangladesh using Oxford Nanopore Minion sequencing equipment at ideSHi.
Timing and spatial scale	Data was collected during March 2020-April 2021.
Data exclusions	Samples which had ct values <31 were excluded from sequencing and the study.
Reproducibility	All methods are fully reproducible according to the detailed instructions in methods and fully accesible sequencing data in Bioproject PRJNA737194.
Randomization	Not applicable.
Blinding	Not applicable.

Did the study involve field work? ☐ Yes ☒ No

Reporting for specific materials, systems and methods

We require information from authors about some types of materials, experimental systems and methods used in many studies. Here, indicate whether each material, system or method listed is relevant to your study. If you are not sure if a list item applies to your research, read the appropriate section before selecting a response.

Materials & experimental systems

n/a	Involved in the study
☒	☐ Antibodies
☒	☐ Eukaryotic cell lines
☒	☐ Palaeontology and archaeology
☒	☐ Animals and other organisms
☒	☐ Human research participants
☐	☒ Clinical data
☒	☐ Dual use research of concern

Methods

n/a	Involved in the study
☒	☐ ChIP-seq
☒	☐ Flow cytometry
☒	☐ MRI-based neuroimaging

Clinical data

Policy information about clinical studies

All manuscripts should comply with the ICMJE guidelines for publication of clinical research and a completed CONSORT checklist must be included with all submissions.

Clinical trial registration	Not applicable.
Study protocol	Not applicable.
Data collection	IEDCR, Dhaka, March 2020-April 2021
Outcomes	Not applicable.
