# Supplementary material for: Genomics, social media and mobile phone data enable mapping of SARS-CoV-2 lineages to inform health policy in Bangladesh
Source: Nat Microbiol. 2021 Sep 8;6(10):1271–8. doi: 10.1038/s41564-021-00955-3 (PMC8478645; doi:10.1038/s41564-021-00955-3)
Supplement: Supplementary file 4 — Peer Review Information [file 41564_2021_955_MOESM4_ESM.pdf]

natureresearch

Peer Review Information

Journal: Nature Microbiology
Manuscript Title: **NMICROBIOL-21010154B**
Corresponding author name(s): Firdausi Qadri

Reviewer Comments & Decisions:

Decision Letter, initial version:

Dear Dr. Qadri,

Thank you very much for your patience while your manuscript "Genomic and mobility data reveal mass population movement as a driver of SARS-CoV-2 dissemination and diversity in Bangladesh" was under peer-review at Nature Microbiology. It has now been seen by 2 referees, whose expertise and comments you will find at the end of this email. Although they find your work of some potential interest, they have raised a number of concerns that will need to be addressed before we can consider publication of the work in Nature Microbiology.

In particular, referee #1 requests to analyse potential sampling biases within the data and to add quality control details from the nanopore sequencing, as well as to clarify several points regarding the Bayesian phylogenetic analysis. This referee also suggests to perform statistical correlations of the mobility data with epidemiology parameters (e.g. prevalence, hospital admissions etc.). We suggest to add a specific "limitations section" to the manuscript that reflects on the limitations of this study and to include the reasoning for the analytic plan in the main text. Please try and address the points raised as swiftly as possible and ensure that the literature for the Introduction and Discussion is completely updated. Please note that in case we decide to send the manuscript back to the referees, we will eventually ask an additional referee to evaluate the manuscript, as one reviewer did not cover all the aspects we were hoping to see covered.

Should further extensive re-analysis as requested by the referees allow you to address these criticisms, we would be happy to look at a revised manuscript. However, we would like to make it clear that we would only engage our referees again for re-evaluation if we feel that their comments have been addressed in full, resulting in a substantial improvement of the manuscript.

We are committed to providing a fair and constructive peer-review process. Please do not hesitate to contact us if there are specific requests from the reviewers that you believe are technically impossible or

unlikely to yield a meaningful outcome.

We strongly support public availability of data. Please place the data used in your paper into a public data repository, if one exists, or alternatively, present the data as Source Data or Supplementary Information. If data can only be shared on request, please explain why in your Data Availability Statement, and also in the correspondence with your editor. For some data types, deposition in a public repository is mandatory - more information on our data deposition policies and available repositories can be found at https://www.nature.com/nature-research/editorial-policies/reporting-standards#availability-of-data.

Please include a data availability statement as a separate section after Methods but before references, under the heading "Data Availability". This section should inform readers about the availability of the data used to support the conclusions of your study. This information includes accession codes to public repositories (data banks for protein, DNA or RNA sequences, microarray, proteomics data etc…), references to source data published alongside the paper, unique identifiers such as URLs to data repository entries, or data set DOIs, and any other statement about data availability. At a minimum, you should include the following statement: "The data that support the findings of this study are available from the corresponding author upon request", mentioning any restrictions on availability. If DOIs are provided, we also strongly encourage including these in the Reference list (authors, title, publisher (repository name), identifier, year). For more guidance on how to write this section please see:
http://www.nature.com/authors/policies/data/data-availability-statements-data-citations.pdf

If revising your manuscript:

* Include a "Response to referees" document detailing, point-by-point, how you addressed each referee comment. If no action was taken to address a point, you must provide a compelling argument. This response will be sent back to the referees along with the revised manuscript.

* If you have not done so already we suggest that you begin to revise your manuscript so that it conforms to our Article format instructions at http://www.nature.com/nmicrobiol/info/final-submission. Refer also to any guidelines provided in this letter.

* Include a revised version of any required reporting checklist. It will be available to referees (and, potentially, statisticians) to aid in their evaluation if the manuscript goes back for peer review. A revised checklist is essential for re-review of the paper.

When submitting the revised version of your manuscript, please pay close attention to our href="https://www.nature.com/nature-research/editorial-policies/image-integrity">Digital Image Integrity Guidelines. and to the following points below:

-- that unprocessed scans are clearly labelled and match the gels and western blots presented in figures.
-- that control panels for gels and western blots are appropriately described as loading on sample processing controls
-- all images in the paper are checked for duplication of panels and for splicing of gel lanes.

Finally, please ensure that you retain unprocessed data and metadata files after publication, ideally archiving data in perpetuity, as these may be requested during the peer review and production process or after publication if any issues arise.

Please use the link below to submit a revised paper:

{REDACTED}

Note: This url links to your confidential homepage and associated information about manuscripts you may have submitted or be reviewing for us. If you wish to forward this e-mail to co-authors, please delete this link to your homepage first.

Nature Microbiology is committed to improving transparency in authorship. As part of our efforts in this direction, we are now requesting that all authors identified as 'corresponding author' on published papers create and link their Open Researcher and Contributor Identifier (ORCID) with their account on the Manuscript Tracking System (MTS), prior to acceptance. This applies to primary research papers only. ORCID helps the scientific community achieve unambiguous attribution of all scholarly contributions. You can create and link your ORCID from the home page of the MTS by clicking on 'Modify my Springer Nature account'. For more information please visit please visit www.springernature.com/orcid.

Due to the fast-moving nature of the SARS-CoV-2/COVID-19 field, we will re-assess novelty of the work at the time of resubmission if you wish to submit a suitably revised manuscript. We would hope to receive it within 2 months. If you cannot send it within this time, please let us know.

In the meantime we hope that you find our referees' comments helpful.

{REDACTED}

****
Reviewer Expertise:

Referee #1: Virus evolution, phylogenies, Emerging viruses
Referee #2: Microbial analysis, Phylogenetics, Population comparisions

Reviewer Comments:

Reviewer #1 (Remarks to the Author):

Cowley et al. analyze SARS-CoV-2 full genome sequences from Bangladesh sampled between March 8 and July 31 2020 (67 new sequences and 324 available in GISAID) to investigate the introduction and initial spread of the virus in the country. Together with the analysis of mobility data, inferred from social media (Facebook Data for Good) and mobile phone call detail records, they conclude that after the introductions of several viral lineages from abroad, during the early phase of the epidemic, population

mobility out of Dhaka and other urban hotspots
resulted in rapid country-wide dissemination. The conclusion is not surprising, since it matches a pattern that has been observed and described in depth in many countries worldwide.

The authors also state that "the data on the genomic changes of SARS-CoV-2 in Bangladesh is reassuring" because they suggest that vaccination scale up could be effective, considering that Bangladesh already has extensive experience of conducting mass vaccination campaigns. Unfortunately, the data and analyses presented in the paper hardly support this claim. The dynamic nature of the pandemic, as observed during the past year, has led to multiple epidemic waves in many countries, as well as the emergence and dissemination of new viral lineages, some of which potentially more transmissible or pathogenic. While here may be a "historical" interest in reconstructing the early phases of the epidemic in Bangladesh, the analysis of a few hundred sequences sampled almost one year ago says very little about the current challenges that may be posed by epidemic spread and affect vaccination interventions.

From a technical standpoint, analytical methods are over simplistic and and not very robust.

Major remarks

1. The Bangladesh sequence data set (491 sequences) may be too limited to capture the actual genetic diversity of the virus in the country, or be representative of major phylogeography patterns. There should have been at leas an attempt to investigate potential sampling bias.

2. Sequencing with Nanopore technology is fast but has also a high error rate. No details are given about sequences quality control or detection and masking of known sites associated with high sequencing errors.

3. The Bayesian phylogenetic analysis misses crucial details to assess its reliability: did the sequence display sufficient temporal and phylogenetic signal for reliable inference? which demographic priors were chosen and why? The authors talk about migration patterns inferred from the tree, but it does not look like they carried out a full Bayesian phylogeography analysis. Also, it has been recently shown (Lemey et al. Nature Comm 2020) that inferring migration from Bayesian (or ML) trees from SARS-CoV-2 sequences without taking into account, at least partially, known travel histories of individual patients can be misleading

4. Conclusions about viral spread by simply inspecting the phylogenetic trees can be highly misleading. In such large trees, most branching patterns are not highly supported by either bootstrapping or posterior probabilities (neither of which are reported in the trees anyway). Most conclusions may just reflect artifacts due to phylogenetic uncertainly.

5. Mobility analysis using social and mobile phone data is interesting, but there is no attempt to investigate any possible statistical correlation with epidemiology parameters (incidence prevalence, hospital admissions, etc.), or with findings based on viral sequence analyses. The qualitative observation of similar trends in sequence based analysis finding and mobility data is very weak unless supported, for example, by matching simulations.

Reviewer #2 (Remarks to the Author):

The paper by Cowley and colleagues presents an investigation aimed to investigate patterns of population mobility in Bangladesh and a phylogenetic analysis on SARS-CoV-2 sequences retrieved from GISAID and 67 novel genomes sequenced for this study. The paper shows how the spread in Bangladesh is linked to repeated importations of the SARS-CoV-2 virus from foreign countries due to international travel and how migrations out of the cities impacted on the pandemic.
Overall, the manuscript is well written with interesting data on mobility inferred using mobile operators and Facebook location data and a time scale of the phylogenetic analysis of all the available SARS-CoV-2 genomes from Bangladesh. I have very few minor remarks.
Line 84: The "o" in SARS-COV-2 should be lowercase.
Line 128: The software is called pangolin (the "p" is not capitalized); in the text sometimes it is referred with or without a capital "p".
Line 179: Besides Ref 8 (Buckee et al., 2016), it may be worth adding a citation to https://doi.org/10.1016/S1473-3099(20)30553-3 which used data derived from mobile operator to visualize trends of mobility in the US.
Line 277: The acronym meaning for ideSHI is missing.
Line 279: The words table and figure should be always capitalized in the text.
Line 285: The number of isolates that acquired SNP C30037T is missing from Supplementary Table 4. Also, I would parenthesize "see Supplementary Table 4 for a breakdown […]".
Line 286: A point is missing before "Bayesian".
Line 288: I would consider adding a citation to support the sentence starting on line 286 since it is missing.
Line 338-340: Is this comparison statically significant?
Figure 1: It was really hard for me to see the importing events mentioned in the text from the tree in panel A. I would consider plotting the tree using a circular cladogram and including the lineage annotation using a colored bar/ring. Also, there are no probability values included in the internal nodes. In my opinion, in addition to panel B, it would help having a figure showing the frequencies of the different lineages over time.
Figure 2A: What are the grey lines below the colored one representing the districts?

Author Rebuttal to Initial comments

Reviewer #1

Cowley et al. analyze SARS-CoV-2 full genome sequences from Bangladesh sampled between March 8 and July 31 2020 (67 new sequences and 324 available in GISAID) to investigate the introduction and initial spread of the virus in the country. Together with the analysis of mobility data, inferred from social media (Facebook Data for Good) and mobile phone call detail records,

they conclude that after the introductions of several viral lineages from abroad, during the early phase of the epidemic, population mobility out of Dhaka and other urban hotspots

resulted in rapid country-wide dissemination. The conclusion is not surprising, since it matches a pattern that has been observed and described in depth in many countries worldwide.

The authors also state that "the data on the genomic changes of SARS-CoV-2 in Bangladesh is reassuring" because they suggest that vaccination scale up could be effective, considering that Bangladesh already has extensive experience of conducting mass vaccination campaigns.

Unfortunately, the data and analyses presented in the paper hardly support this claim. The dynamic nature of the pandemic, as observed during the past year, has led to multiple epidemic waves in many countries, as well as the emergence and dissemination of new viral lineages, some of which potentially more transmissible or pathogenic. While here may be a "historical" interest in reconstructing the early phases of the epidemic in Bangladesh, the analysis of a few hundred sequences sampled almost one year ago says very little about the current challenges that may be posed by epidemic spread and affect vaccination interventions.

We agree that the manuscript needed updating and put into the context of recent emergence of variants of concern. Consequently, we have substantially updated the manuscript to include our recent results on the dominance of B.1.351 in Bangladesh in the first quarter of 2021. We have removed all mention of assurance of the success of the vaccine campaign and cautioned policymakers on the risk factors and contributors to the dissemination of SARS-CoV-2 in Bangladesh in early-mid 2020 that could similarly be applied to the current epidemic that is dominated by lineage B.1.351. We believe this addition to the manuscript brings it up to date and heightens the impact and applicability of our reconstruction of the first wave to inform decisions being made in the management of B.1.351 and the implications for the current Oxford Aztrazeneca vaccine administration.

From a technical standpoint, analytical methods are over simplistic and not very robust.

We are very sorry that the reviewer viewed the original manuscript in this way but are confident that the addition and clarifications that we have added into the manuscript (detailed below) will convince them of the robustness of our results.

Major remarks

***Comment 1**. The Bangladesh sequence data set (491 sequences) may be too limited to capture the actual genetic diversity of the virus in the country or be representative of major phylogeography patterns. There should have been at least an attempt to investigate potential sampling bias.*

Thanks to the reviewer for raising this very important issue. We would like to address the comment as follows:

- The initial study was conducted between April 2020 to August 2020 when the pandemic situation due to SARS-CoV-2 was at the highest peak in Bangladesh. There was limited opportunity to do real-time genomic surveillance due to the limitations of the country wide health management system and also due to lack of availability of reagents for the genomics studies and the limitations in procuring these and shipment to Bangladesh since international flights were mostly suspended. Also, movement within Bangladesh had become difficult. With limited resources available, we attempted to accumulate random samples using all our efforts with international collaborators and with support of the Government of Bangladesh.
- To maximise nationwide diversity sampling, we actively included samples from all over the country.
- Multiple studies results were included in our analysis. To provide context for our 65 sequenced samples from the first wave, we used 324 publicly available sequences produced by other groups in Bangladesh (gratefully acknowledged in supplementary material). Given that each study group had access to different samples, this pooling of samples from across multiple studies provides a level of stochasticity and randomness to the sampling that should diminish the risk of sampling bias that can occur when all samples were collected by the same study.
- Additionally, the limitations of amplicon sequencing and the necessity for amplifiable samples further limits our ability to increase the sampling size. We had access to more samples but these had high Ct values by RT-PCR and we were unable to amplify enough DNA product for sequencing. Since we used minion sequencing platform, we only selected those with Ct value less than 30.

We have updated the manuscript to include a limitations section (lines 435-444) that discusses and highlights the very low sample size and the limitations associated with that. We feel that sequencing sample sizes will always be smaller in countries limited by resources (e.g. LMICs) but that so long as appropriate mitigation of sampling bias is attempted (as outlined above) that these small samples are still highly important and informative to the global response to COVID-

19.

Comment 2. *Sequencing with Nanopore technology is fast but has also a high error rate. No details are given about sequences quality control or detection and masking of known sites associated with high sequencing errors.*

This is an important point and we are grateful for the opportunity to clarify how we performed quality control for our nanopore sequencing, as follows:

- We only used passed reads in our analysis which have a high base quality threshold. We also used medaka for sequence correction, variant calling and consensus sequence generation. We used a higher coverage (200x) than standardized for Illumina sequencing (30x) to account for random sequencing errors.
- Consensus sequences were generated by mapping to the Wuhan reference genome and regions with low coverage (<200x) or low quality were masked with Ns.

We have updated this on lines 237-242 of the manuscript.

Comment 3. *The Bayesian phylogenetic analysis misses crucial details to assess its reliability: did the sequence display sufficient temporal and phylogenetic signal for reliable inference? which demographic priors were chosen and why? The authors talk about migration patterns inferred from the tree, but it does not look like they carried out a full Bayesian phylogeography analysis. Also, it has been recently shown (Lemey et al. Nature Comm 2020) that inferring migration from Bayesian (or ML) trees from SARS-CoV-2 sequences without taking into account, at least partially, known travel histories of individual patients can be misleading*

We appreciate the reviewers suggestions and have provided further detail on our Bayesian phylogenetic analysis in the manuscript and here:

- We used TempEst v.1.5.3 to investigate the evolutionary tempo of the Bangladesh SARS-CoV-2 samples. We found a positive correlation with temporal signal and a low to moderate association between genetic distances and sampling dates (R^2=0.2471), reflecting the low mutation rate of SARS-CoV-2, which is consistent with findings reported elsewhere. Given the positive correlation, we determined this data was suitable for phylogenetic molecular clock analysis in BEAST. This has been included in lines 258-263 of the manuscript and the below figure has been included in

supplementary materials (supplementary figure 1).

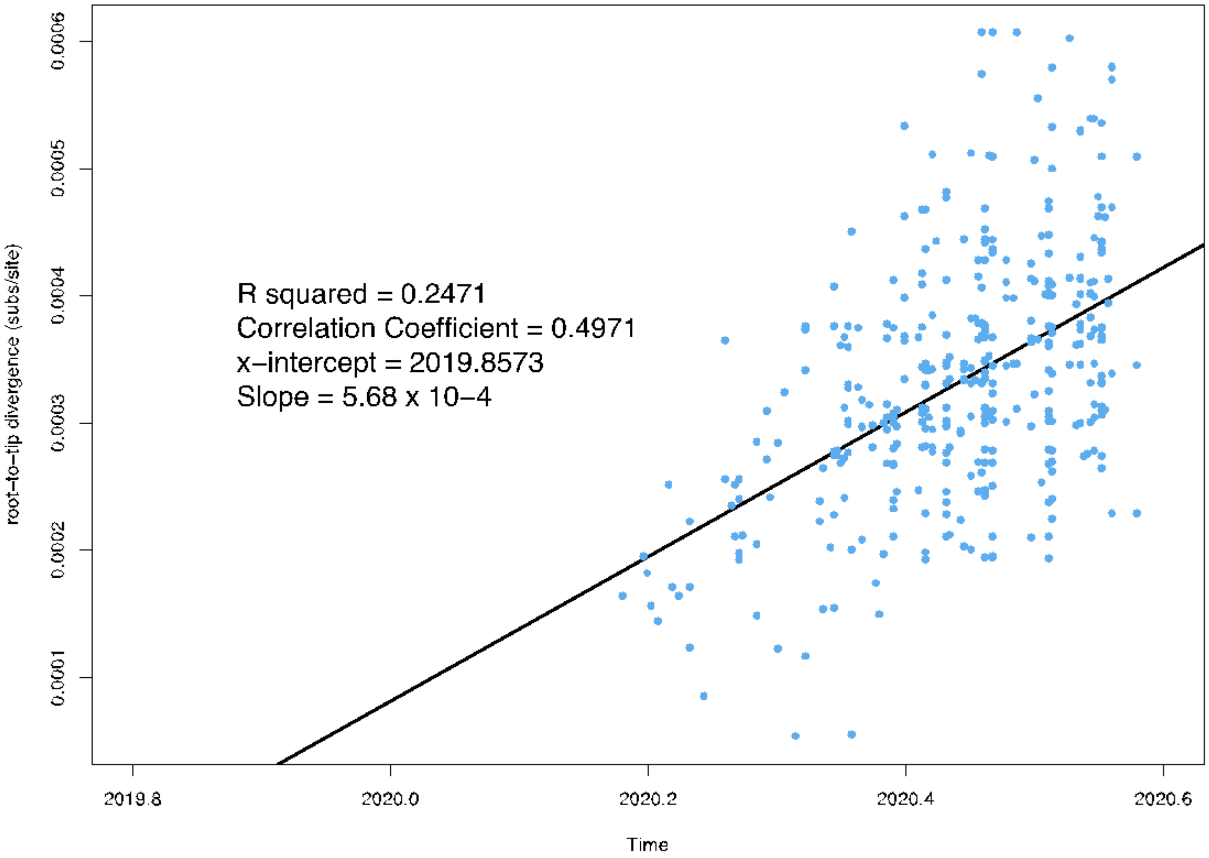

- As we had observed a linear trend without large residual variance, we used a strict molecular clock with the HKY+Γ model of nucleotide substitution. We also used a time-aware coalescent Bayesian exponential growth model available in BEAST (v 1.10.4) and an informative prior reflecting recent estimates for the substitution rate of SARS-CoV-2.

To account for misleading conclusions based solely on phylogenetic data, we used travel history epidemiological metadata to investigate the importation of lineages. We were able to link the importation of lineage B.1 at the beginning of the outbreak to a returning traveler from Italy. Additionally, we linked the importation of lineage B.1.36 to a returning traveler from Saudi Arabia. As well as returning travelers from Saudi Arabia and Italy we also sequenced cases from a returnee from the USA and their contacts. We also used international flight arrival travel data, as detailed in figure 1, to inform the most travelled routes into Bangladesh.

Comment 4. *Conclusions about viral spread by simply inspecting the phylogenetic trees can be highly misleading. In such large trees, most branching patterns are not highly supported by either bootstrapping or posterior probabilities (neither of which are reported in the trees anyway). Most conclusions may just reflect artifacts due to phylogenetic uncertainly.*

We have been extremely cautious to not over-interpret our data and consequently, our conclusions are fairly modest and based mostly on the combination of genomic lineage assignment and mobility data. Our main conclusion was that the national general holiday induced a large proportion of the Dhaka population to return to rural areas of the country which resulted in the expansion of three lineages. This conclusion could be drawn without even assessing the phylogeny of the Bangladeshi cases and based solely on Pango lineage assignment. We have included an additional figure on this in the manuscript (new Figure 2 in the manuscript and displayed below) to clarify the data underlying the expansion and dispersion of the three major lineages. This is detailed in the new figure as follows:

- Panels A and B depict the diversity of lineages present in March and how just three (B.1.1, B.1.1.25 and B.1.36) become increasingly dominant between April and July 2020.
- Panel C depicts the geographic spread of those lineages and how their incidence moves from Dhaka to the other divisions following the National General Holiday in Bangladesh at the end of March.

The mobility data from facebook and three mobile phone operators, as shown in figure 3, provides demographic evidence on how this dispersion and expansion of the three major lineages occurred. There is a clear migration from Dhaka to regional areas which transposed lineages imported at major international hubs (such as Dhaka and Chattogram) into regional areas when workers returned home for the National General Holiday.

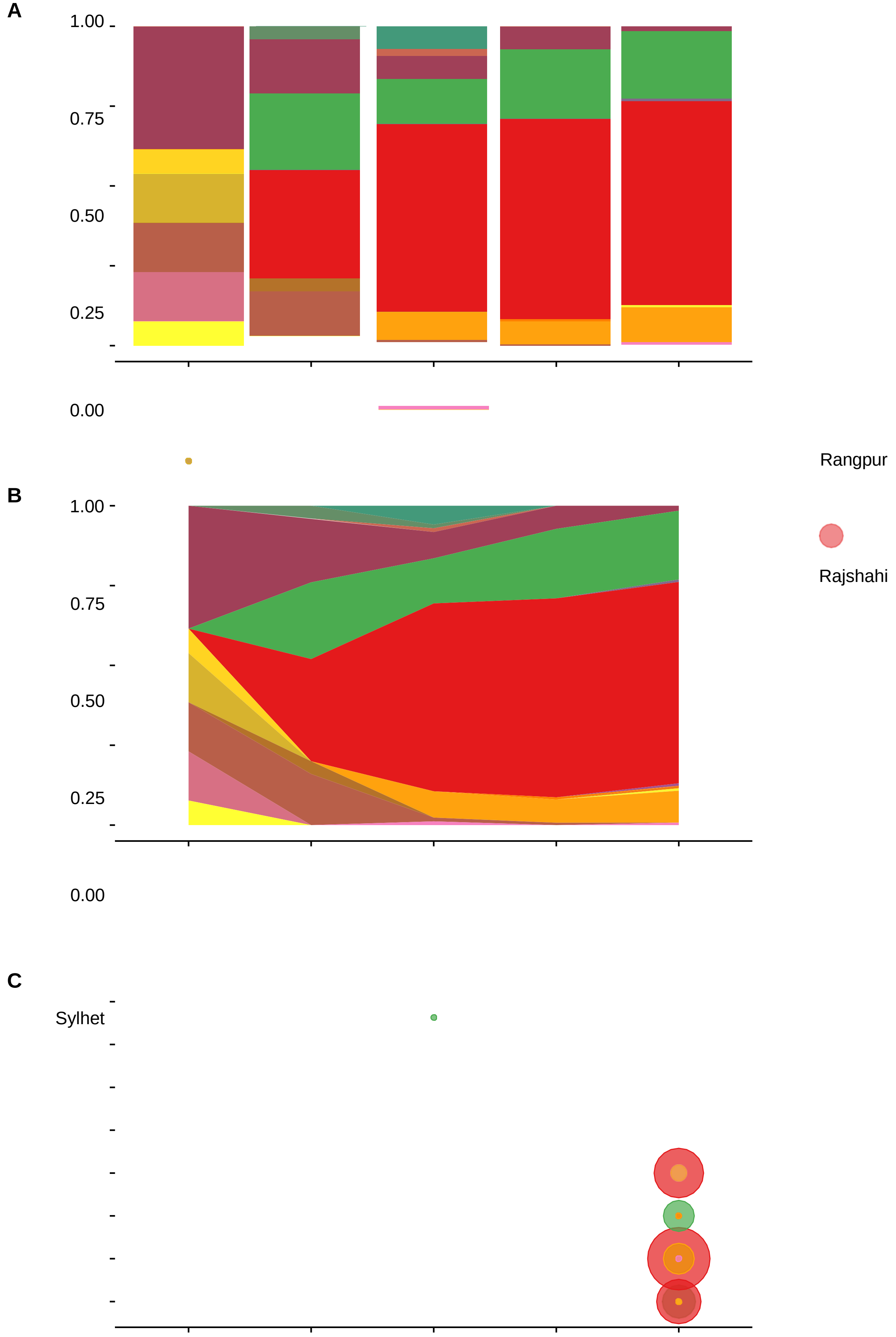
A
1.00
0.75
0.50
0.25
0.00
Rangpur
B
1.00
0.75
0.50
0.25
0.00
Rajshahi
C
Sylhet

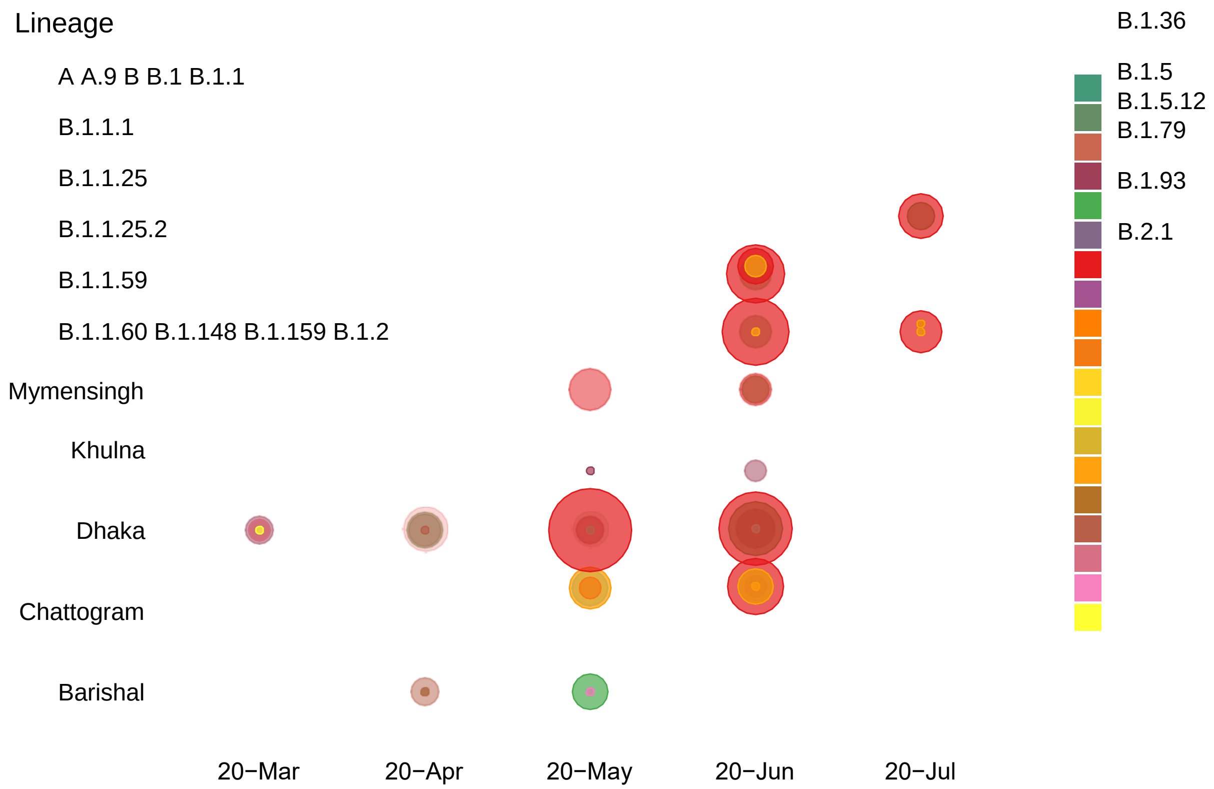
Lineage
A A.9 B B.1 B.1.1
B.1.1.1
B.1.1.25
B.1.1.25.2
B.1.1.59
B.1.1.60 B.1.148 B.1.159 B.1.2
B.1.36
B.1.5
B.1.5.12
B.1.79
B.1.93
B.2.1
Mymensingh
Khulna
Dhaka
Chattogram
Barishal
20−Mar
20−Apr
20−May
20−Jun
20−Jul

***Comment 5.** Mobility analysis using social and mobile phone data is interesting, but there is no attempt to investigate any possible statistical correlation with epidemiology parameters (incidence prevalence, hospital admissions, etc.), or with findings based on viral sequence analyses. The qualitative observation of similar trends in sequence based analysis finding and mobility data is very weak unless supported, for example, by matching simulations.*

Given the paucity of surveillance data on the epidemic – particularly cases and reporting rates – we feel that it would not make sense to attempt to statistically compare the mobility data with other information, which is why we chose a qualitative approach. In particular, the epidemiological parameters necessary to do such analyses are likely to show time and space varying uncertainty that was unmeasured, and even if inferred through a full mechanistic model, would be impossible to validate or make sense of. There are nowcasting approaches that use death rate data to estimate transmission parameters (see McGough et al., 2020, PLoS Comp Bio), but here, too, spatial and temporal resolution of accurate death data is needed in order to accurately reconstruct epidemic dynamics, and goes beyond the scope of this manuscript. We wanted to avoid the mistake of erroneously fitting a mechanistic simulation model of SARS-CoV-2 transmission given these well-documented uncertainties. We note that statistical significance per se is not necessarily informative with respect to mobile phone data in general, because the sample sizes are so large that spurious significance is easy to show. Instead, we find the consistency between two completely different indicators of the epidemic from genomics and mobility data to be strongly supportive of our conclusions.

Reviewer #2:

The paper by Cowley and colleagues presents an investigation aimed to investigate patterns of population mobility in Bangladesh and a phylogenetic analysis on SARS-CoV-2 sequences retrieved from GISAID and 67 novel genomes sequenced for this study. The paper shows how the spread in Bangladesh is linked to repeated importations of the SARS-CoV-2 virus from foreign countries due to international travel and how migrations out of the cities impacted on the pandemic.

Overall, the manuscript is well written with interesting data on mobility inferred using mobile operators and Facebook location data and a time scale of the phylogenetic analysis of all the available SARS-CoV-2 genomes from Bangladesh. I have very few minor remarks.

We would like to thank the reviewer for their careful consideration of our manuscript, we appreciate their suggestions and corrections and have resolved all of them as detailed below.

Comments:

Comment 1: Line 84: The "o" in SARS-COV-2 should be lowercase.

Corrected.

Comment 2: Line 128: The software is called pangolin (the "p" is not capitalized); in the text sometimes, it is referred with or without a capital "p".

Corrected.

Comment 3: Line 179: Besides Ref 8 (Buckee et al., 2016), it may be worth adding a citation to https://doi.org/10.1016/S1473-3099 [linkprotect.cudasvc.com] (20)30553-3 which used data derived from mobile operator to visualize trends of mobility in the US.

Thanks for the suggestion, re-cited as suggested.

Comment 4: Line 277: The acronym meaning for ideSHI is missing.

Corrected. Now the new line 314-316 reads,
Samples were tested for SARS-CoV-2 using RT-PCR and 67 positive samples were sequenced at the Institute for Developing Science and Health Initiatives (ideSHi) for sequencing.

Comment 5: Line 279: The words table and figure should be always capitalized in the text.

Corrected.

Comment 6: Line 285: The number of isolates that acquired SNP C30037T is missing from Supplementary Table 4. Also, I would parenthesize "see Supplementary Table 4 for a breakdown [...]".

Thanks for pointing out. We have included all the non-synonymous SNP in the new updated Supplementary Table 3. This SNP C30037T is silent (variant: F106F present in NSP3) and this is the reason we didn't include this SNP in the supplementary table. We also parenthesize Supplementary Table 3 in the text as the reviewer suggested.

Comment 7: Line 286: A point is missing before "Bayesian".

We are unclear what the reviewer meant here as Bayesian is the first word of a sentence.

Comment 8: Line 288: I would consider adding a citation to support the sentence starting on line 286 since it is missing.

Citation now included.

Comment 9: Line 338-340: Is this comparison statically significant?

The following sentence has been inserted in lines 380-384. The percentage of long-distance trips (>50 km) traveled in the month of Eid (July) was significantly different from that of other months; while the percentage of long-distance trips traveled in July was 71.1% (95% CI: 71.0%- 71.2%), the same proportion in August, the next highest month, was 57.9% (95% CI: 57.9%- 58.0%).

Comment 10: Figure 1: It was really hard for me to see the importing events mentioned in the text from the tree in panel A. I would consider plotting the tree using a circular cladogram and including the lineage annotation using a colored bar/ring. Also, there are no probability values included in the internal nodes. In my opinion, in addition to panel B, it would help having a figure showing the frequencies of the different lineages over time.

Two new figures have been added to the manuscript that should make the lineage dynamics

and phylogeography results clearer to the reader. New figure 2, provides a frequency barplot of the different lineages over time as suggested and new figure 4 provides a clearer tree that clearly shows the number of times that key lineages have been imported into Bangladesh.

Comment 11: Figure 2A: What are the grey lines below the colored one representing the districts?

Those grey lines represent the different district of Bangladesh, the figure legend has been updated to clarify this.

Decision Letter, first revision:

Dear Dr. Qadri,

Thank you for submitting your revised manuscript "Shifting lineage dynamics of SARS-CoV-2 in Bangladesh; driven by population mobility in the first wave and the dominance of B.1.351 in the second wave" (NMICROBIOL-21010154A). It has now been seen by the original referees and their comments are below. The reviewers find that the paper has improved in revision, and therefore we'll be happy in principle to publish it in Nature Microbiology, pending minor revisions to satisfy the referees' final requests and to comply with our editorial and formatting guidelines. Thanks also for your note and letter, please let me know whether you would be willing to add a short summary for 'policy implications' in the Discussion.

If the current version of your manuscript is in a PDF format, please email us a copy of the file in an editable format (Microsoft Word or LaTex)-- we can not proceed with PDFs at this stage.

We are now performing detailed checks on your paper and will send you a checklist detailing our editorial and formatting requirements in about a week. Please do not upload the final materials and make any revisions until you receive this additional information from us.

Thank you again for your interest in Nature Microbiology Please do not hesitate to contact me if you have any questions.

{REDACTED}

Reviewer #1 (Remarks to the Author):

The revised version of the manuscript includes additional sequences for the first quarter of 2021 showing emergence and dominance of variant B.1.351 in Bangladesh. I appreciate the authors effort to make the manuscript more informative about the current situation in the country by discussing recent data. Unfortunately, the methodological limitations have not been addressed. The major problem remains that the limited amount of sequences generated and analyze are unlikely to be

representative of general SARS-CoV-2 variant dynamics in the country. We appreciate the difficulty, especially at the beginning of the epidemic, for real time genomic tracking. Nevertheless, the authors do not even attempt to evaluate potential sampling bias in the data set or use a more sophisticated analytical approach that may assess the impact of missing data (e.g hidden Markov modeling). Therefore, the results displayed can at best be indicative of a trend in variants distribution shifting over time, which has been already reported observed in several studies in different countries and it is, therefore, of limited interest.

Additional major remarks:

1. Assessing importation of specific lineages using patients travel histories is, again, dependent, on representative sampling and reassurance of no missing transmission links, which the authors cannot provide.

2.Problems about the reliability and interpretation of the phylogeny also have not been addressed. Combination of genomic lineage assignment and mobility data is based on qualitative comparison of changes over time, and it is not sufficiently robust to draw firm conclusions, aside the obvious consideration (as demonstrated in may other studies) that travel impacts epidemic spread.

Reviewer #2 (Remarks to the Author):

The authors adequately addressed all the raised concerns. I do not have further comments.

Reviewer #3 (Remarks to the Author):

In this study, the authors compare information extracted from genomic sequence data and from anonymized mobility data to characterize the early phase of the COVID-19 pandemic in Bangladesh. Both datasets point to a consistent dissemination pattern of SARS-CoV-2, driven by population mobility from Dakha and other urban centers to the rest of the country.

Here, I focus my comments on the analyses of mobility data which is the part of the manuscript I am most confident to assess.

As a standalone, the analysis of mobility data is not particularly innovative since the availability of such data streams has become common.
Indeed, since the start of the COVID-19 pandemic, the type of mobility data used in the manuscript has become a standard to assess population movements in response to the outbreak.
On a more positive side, the comparison of Facebook mobility data with CDRs derived population flows represents a good check of consistency since the quality of Facebook data in a resource-poor setting like Bangladesh is not obvious.
Also, the collection and analysis of such data streams in Bangladesh represent an excellent example of the public health value of mobile phone data in LMICs.

The added value of the study would mostly come from the combination of mobility data with genomic data.
However, as noted by other referees, the comparison is purely qualitative and does not rely on

statistical modeling.
I agree with the authors when they say that developing a mechanistic model of SARS-CoV-2 spread incorporating human movements would go beyond the scope of the study, and it would require much better epidemiological data for calibration.

However, in the literature, there are already studies that integrate these types of data in a more systematic and statistically solid way.
For instance, consider the example of:

Lemey, Philippe, et al. "SARS-CoV-2 European resurgence foretold: interplay of introductions and persistence by leveraging genomic and mobility data." (2021).
https://pubmed.ncbi.nlm.nih.gov/33594355/

where mobility data are used to fit a Bayesian phylogeographic model.

Or the example of Lemey, Philippe, et al. "Accommodating individual travel history and unsampled diversity in Bayesian phylogeographic inference of SARS-CoV-2." Nature communications 11.1 (2020): 1-14. where individual travel history data are combined with mobility data to fit a similar model.

I believe that a similar analysis (especially in line with the approach of the first reference I mentioned) would significantly strengthen the manuscript with respect to its current form.

Decision Letter, final checks:

Dear Dr. Qadri and Dr Cowley,

I am sorry this email did not reach you when it was sent on the 12th of July. We are sending it through a second time.

Thank you for your patience as we've prepared the guidelines for final submission of your Nature Microbiology manuscript, "Shifting lineage dynamics of SARS-CoV-2 in Bangladesh; driven by population mobility in the first wave and the dominance of B.1.351 in the second wave" (NMICROBIOL-21010154A). Please carefully follow the step-by-step instructions provided in the attached file, and add a response in each row of the table to indicate the changes that you have made. Please also check and comment on any additional marked-up edits we have proposed within the text. Ensuring that each point is addressed will help to ensure that your revised manuscript can be swiftly handed over to our production team.

We would like to start working on your revised paper, with all of the requested files and forms, as soon as possible (preferably within two weeks). Please get in contact with us if you anticipate delays.

When you upload your final materials, please include a point-by-point response to any remaining reviewer comments.

If you have not done so already, please alert us to any related manuscripts from your group that are

under consideration or in press at other journals, or are being written up for submission to other journals (see: https://www.nature.com/nature-research/editorial-policies/plagiarism#policy-on-duplicate-publication for details).

In recognition of the time and expertise our reviewers provide to Nature Microbiology's editorial process, we would like to formally acknowledge their contribution to the external peer review of your manuscript entitled "Shifting lineage dynamics of SARS-CoV-2 in Bangladesh; driven by population mobility in the first wave and the dominance of B.1.351 in the second wave". For those reviewers who give their assent, we will be publishing their names alongside the published article.

Nature Microbiology offers a Transparent Peer Review option for new original research manuscripts submitted after December 1st, 2019. As part of this initiative, we encourage our authors to support increased transparency into the peer review process by agreeing to have the reviewer comments, author rebuttal letters, and editorial decision letters published as a Supplementary item. When you submit your final files please clearly state in your cover letter whether or not you would like to participate in this initiative. Please note that failure to state your preference will result in delays in accepting your manuscript for publication.

Cover suggestions

As you prepare your final files we encourage you to consider whether you have any images or illustrations that may be appropriate for use on the cover of Nature Microbiology.

Covers should be both aesthetically appealing and scientifically relevant, and should be supplied at the best quality available. Due to the prominence of these images, we do not generally select images featuring faces, children, text, graphs, schematic drawings, or collages on our covers.

We accept TIFF, JPEG, PNG or PSD file formats (a layered PSD file would be ideal), and the image should be at least 300ppi resolution (preferably 600-1200 ppi), in CMYK colour mode.

If your image is selected, we may also use it on the journal website as a banner image, and may need to make artistic alterations to fit our journal style.

Please submit your suggestions, clearly labeled, along with your final files. We'll be in touch if more information is needed.

Nature Microbiology has now transitioned to a unified Rights Collection system which will allow our Author Services team to quickly and easily collect the rights and permissions required to publish your work. Approximately 10 days after your paper is formally accepted, you will receive an email in providing you with a link to complete the grant of rights. If your paper is eligible for Open Access, our Author Services team will also be in touch regarding any additional information that may be required to arrange payment for your article.

Please note that you will not receive your proofs until the publishing agreement has been received through our system.

Please note that <i>Nature Microbiology</i> is a Transformative Journal (TJ). Authors may publish

their research with us through the traditional subscription access route or make their paper immediately open access through payment of an article-processing charge (APC). Authors will not be required to make a final decision about access to their article until it has been accepted. Find out more about Transformative Journals

<B>Authors may need to take specific actions to achieve compliance with funder and institutional open access mandates. For submissions from January 2021, if your research is supported by a funder that requires immediate open access (e.g. according to Plan S principles) then you should select the gold OA route, and we will direct you to the compliant route where possible. For authors selecting the subscription publication route our standard licensing terms will need to be accepted, including our self-archiving policies. Those standard licensing terms will supersede any other terms that the author or any third party may assert apply to any version of the manuscript.

For information regarding our different publishing models please see our Transformative Journals page. If you have any questions about costs, Open Access requirements, or our legal forms, please contact ASJournals@springernature.com.

Please use the following link for uploading these materials:

{REDACTED}

If you have any further questions, please feel free to contact me.

{REDACTED}

Reviewer #1:
Remarks to the Author:
The revised version of the manuscript includes additional sequences for the first quarter of 2021 showing emergence and dominance of variant B.1.351 in Bangladesh. I appreciate the authors effort to make the manuscript more informative about the current situation in the country by discussing recent data. Unfortunately, the methodological limitations have not been addressed. The major problem remains that the limited amount of sequences generated and analyze are unlikely to be representative of general SARS-CoV-2 variant dynamics in the country. We appreciate the difficulty, especially at the beginning of the epidemic, for real time genomic tracking. Nevertheless, the authors do not even attempt to evaluate potential sampling bias in the data set or use a more sophisticated analytical approach that may assess the impact of missing data (e.g hidden Markov modeling). Therefore, the results displayed can at best be indicative of a trend in variants distribution shifting over time, which has been already reported observed in several studies in different countries and it is,

therefore, of limited interest.

Additional major remarks:

1. Assessing importation of specific lineages using patients travel histories is, again, dependent, on representative sampling and reassurance of no missing transmission links, which the authors cannot provide.

2.Problems about the reliability and interpretation of the phylogeny also have not been addressed. Combination of genomic lineage assignment and mobility data is based on qualitative comparison of changes over time, and it is not sufficiently robust to draw firm conclusions, aside the obvious consideration (as demonstrated in may other studies) that travel impacts epidemic spread.

Reviewer #2:
Remarks to the Author:
The authors adequately addressed all the raised concerns. I do not have further comments.

Reviewer #3:
Remarks to the Author:
In this study, the authors compare information extracted from genomic sequence data and from anonymized mobility data to characterize the early phase of the COVID-19 pandemic in Bangladesh. Both datasets point to a consistent dissemination pattern of SARS-CoV-2, driven by population mobility from Dakha and other urban centers to the rest of the country.

Here, I focus my comments on the analyses of mobility data which is the part of the manuscript I am most confident to assess.

As a standalone, the analysis of mobility data is not particularly innovative since the availability of such data streams has become common.
Indeed, since the start of the COVID-19 pandemic, the type of mobility data used in the manuscript has become a standard to assess population movements in response to the outbreak.
On a more positive side, the comparison of Facebook mobility data with CDRs derived population flows represents a good check of consistency since the quality of Facebook data in a resource-poor setting like Bangladesh is not obvious.
Also, the collection and analysis of such data streams in Bangladesh represent an excellent example of the public health value of mobile phone data in LMICs.

The added value of the study would mostly come from the combination of mobility data with genomic data.
However, as noted by other referees, the comparison is purely qualitative and does not rely on statistical modeling.
I agree with the authors when they say that developing a mechanistic model of SARS-CoV-2 spread incorporating human movements would go beyond the scope of the study, and it would require much better epidemiological data for calibration.

However, in the literature, there are already studies that integrate these types of data in a more systematic and statistically solid way.
For instance, consider the example of:

Lemey, Philippe, et al. "SARS-CoV-2 European resurgence foretold: interplay of introductions and persistence by leveraging genomic and mobility data." (2021).
https://pubmed.ncbi.nlm.nih.gov/33594355/

where mobility data are used to fit a Bayesian phylogeographic model.

Or the example of Lemey, Philippe, et al. "Accommodating individual travel history and unsampled diversity in Bayesian phylogeographic inference of SARS-CoV-2." Nature communications 11.1 (2020): 1-14. where individual travel history data are combined with mobility data to fit a similar model.

I believe that a similar analysis (especially in line with the approach of the first reference I mentioned) would significantly strengthen the manuscript with respect to its current form.

Final Decision Letter:

Dear Dr Qadri,

I am pleased to accept your Article "Genomics, social media and mobile phone data enable mapping of SARS-CoV-2 lineages to inform health policy in Bangladesh" for publication in Nature Microbiology. Thank you for having chosen to submit your work to us and many congratulations.

Before your manuscript is typeset, we will edit the text to ensure it is intelligible to our wide readership and conforms to house style. We look particularly carefully at the titles of all papers to ensure that they are relatively brief and understandable.

Once your manuscript is typeset and you have completed the appropriate grant of rights, you will receive a link to your electronic proof via email with a request to make any corrections within 48 hours. If, when you receive your proof, you cannot meet this deadline, please inform us at rjsproduction@springernature.com immediately. Once your paper has been scheduled for online publication, the Nature press office will be in touch to confirm the details.

Acceptance of your manuscript is conditional on all authors' agreement with our publication policies (see www.nature.com/nmicrobiolate/authors/gta/content-type/index.html). In particular your manuscript must not be published elsewhere and there must be no announcement of the work to any media outlet until the publication date (the day on which it is uploaded onto our website).

Please note that <i>Nature Microbiology</i> is a Transformative Journal (TJ). Authors may publish their research with us through the traditional subscription access route or make their paper immediately open access through payment of an article-processing charge (APC). Authors will not be required to make a final decision about access to their article until it has been accepted. Find out more about Transformative Journals

<B>Authors may need to take specific actions to achieve compliance with funder and institutional open access mandates. For submissions from January 2021, if your research is supported by a funder that requires immediate open access (e.g. according to Plan S principles) then you should select the gold OA route, and we will direct you to the compliant route where possible. For authors selecting the subscription publication route our standard licensing terms will need to be accepted, including our self-archiving policies. Those standard licensing terms will supersede any other terms that the author or any third party may assert apply to any version of the manuscript.

In a few business days you will receive an email with a link to choose the appropriate publishing options for your paper and our Author Services team will be in touch regarding any additional information that may be required.

You will not receive your proofs until the publishing agreement has been received through our system.

If you have any questions about our publishing options, costs, Open Access requirements, or our legal forms, please contact ASJournals@springernature.com

An online order form for reprints of your paper is available at https://www.nature.com/reprints/author-reprints.html. All co-authors, authors' institutions and authors' funding agencies can order reprints using the form appropriate to their geographical region.

We welcome the submission of potential cover material (including a short caption of around 40 words) related to your manuscript; suggestions should be sent to Nature Microbiology as electronic files (the image should be 300 dpi at 210 x 297 mm in either TIFF or JPEG format). Please note that such pictures should be selected more for their aesthetic appeal than for their scientific content, and that colour images work better than black and white or grayscale images. Please do not try to design a cover with the Nature Microbiology logo etc., and please do not submit composites of images related to your work. I am sure you will understand that we cannot make any promise as to whether any of your suggestions might be selected for the cover of the journal.

You can now use a single sign-on for all your accounts, view the status of all your manuscript submissions and reviews, access usage statistics for your published articles and download a record of your refereeing activity for the Nature journals.

To assist our authors in disseminating their research to the broader community, our SharedIt initiative provides you with a unique shareable link that will allow anyone (with or without a subscription) to read the published article. Recipients of the link with a subscription will also be able to download and print the PDF.

As soon as your article is published, you will receive an automated email with your shareable link.

Your article will also be included in the Springernature COVID-19 collection.
